# Aetiology and outcome in hospitalized cardiac arrest patients

**DOI:** 10.1093/ehjopen/oead066

**Published:** 2023-06-22

**Authors:** Malin Albert, Johan Herlitz, Araz Rawshani, Sune Forsberg, Mattias Ringh, Jacob Hollenberg, Andreas Claesson, Meena Thuccani, Peter Lundgren, Martin Jonsson, Per Nordberg

**Affiliations:** Department of Clinical Science and Education, Södersjukhuset, Centre for Resuscitation Science, Karolinska Institutet, Sjukhusbacken 10, 118 83 Stockholm, Sweden; Centre for Prehospital Research, Faculty of Caring Science, Work Life and Social Welfare, University of Borås, SE-501 90 Borås, Sweden; Department of Molecular and Clinical Medicine, Institute of Medicine, University of Gothenburg, Gothenburg, Sweden; Department of Clinical Science and Education, Södersjukhuset, Centre for Resuscitation Science, Karolinska Institutet, Sjukhusbacken 10, 118 83 Stockholm, Sweden; Department of Clinical Science and Education, Södersjukhuset, Centre for Resuscitation Science, Karolinska Institutet, Sjukhusbacken 10, 118 83 Stockholm, Sweden; Department of Clinical Science and Education, Södersjukhuset, Centre for Resuscitation Science, Karolinska Institutet, Sjukhusbacken 10, 118 83 Stockholm, Sweden; Department of Clinical Science and Education, Södersjukhuset, Centre for Resuscitation Science, Karolinska Institutet, Sjukhusbacken 10, 118 83 Stockholm, Sweden; Department of Anaesthesiology and Intensive Care Medicine, Institute of Clinical Sciences, Sahlgrenska Academy, University of Gothenburg, Gothenburg, Sweden; Centre for Prehospital Research, Faculty of Caring Science, Work Life and Social Welfare, University of Borås, SE-501 90 Borås, Sweden; Department of Molecular and Clinical Medicine, Institute of Medicine, University of Gothenburg, Gothenburg, Sweden; Department of Cardiology, Region Västra Götaland, Sahlgrenska University Hospital, Gothenburg, Sweden; Department of Clinical Science and Education, Södersjukhuset, Centre for Resuscitation Science, Karolinska Institutet, Sjukhusbacken 10, 118 83 Stockholm, Sweden; Department of Clinical Science and Education, Södersjukhuset, Centre for Resuscitation Science, Karolinska Institutet, Sjukhusbacken 10, 118 83 Stockholm, Sweden; Functional Perioperative Medicine and Intensive Care, Karolinska University Hospital, Stockholm, Sweden

**Keywords:** IHCA, CPR, AED, Aetiology

## Abstract

**Aims:**

To study aetiologies of in-hospital cardiac arrests (IHCAs) and their association with 30-day survival.

**Methods and results:**

Observational study with data from national registries. Specific aetiologies (*n* = 22) of IHCA patients between April 2018 and December 2020 were categorized into cardiac vs. non-cardiac and six main aetiology categories: myocardial ischemia, other cardiac causes, pulmonary causes, infection, haemorrhage, and other non-cardiac causes. Main endpoints were proportions in each aetiology, 30-day survival, and favourable neurological outcome (Cerebral Performance Category scale 1–2) at discharge. Among, 4320 included IHCA patients (median age 74 years, 63.1% were men), approximate 50% had cardiac causes with a 30-day survival of 48.4% compared to 18.7% among non-cardiac causes (*P* < 0.001). The proportion in each category were: myocardial ischemia 29.9%, pulmonary 21.4%, other cardiac causes 19.6%, other non-cardiac causes 11.6%, infection 9%, and haemorrhage 8.5%. The odds ratio (OR) for 30-day survival compared to myocardial ischemia for each category were: other cardiac causes OR 1.48 (CI 1.24–1.76); pulmonary causes OR 0.36 (CI 0.3–0.44); infection OR 0.25 (CI 0.18–0.33); haemorrhage OR 0.22 (CI 0.16–0.3); and other non-cardiac causes OR 0.56 (CI 0.45–0.69). IHCA caused by myocardial ischemia had the best favourable neurological outcome while those caused by infection had the lowest OR 0.06 (CI 0.03–0.13).

**Conclusion:**

In this nationwide observational study, aetiologies with cardiac and non-cardiac causes of IHCA were evenly distributed. IHCA caused by myocardial ischemia and other cardiac causes had the strongest associations with 30-day survival and neurological outcome.

## Graphical Abstract

**Graphical Abstract:**
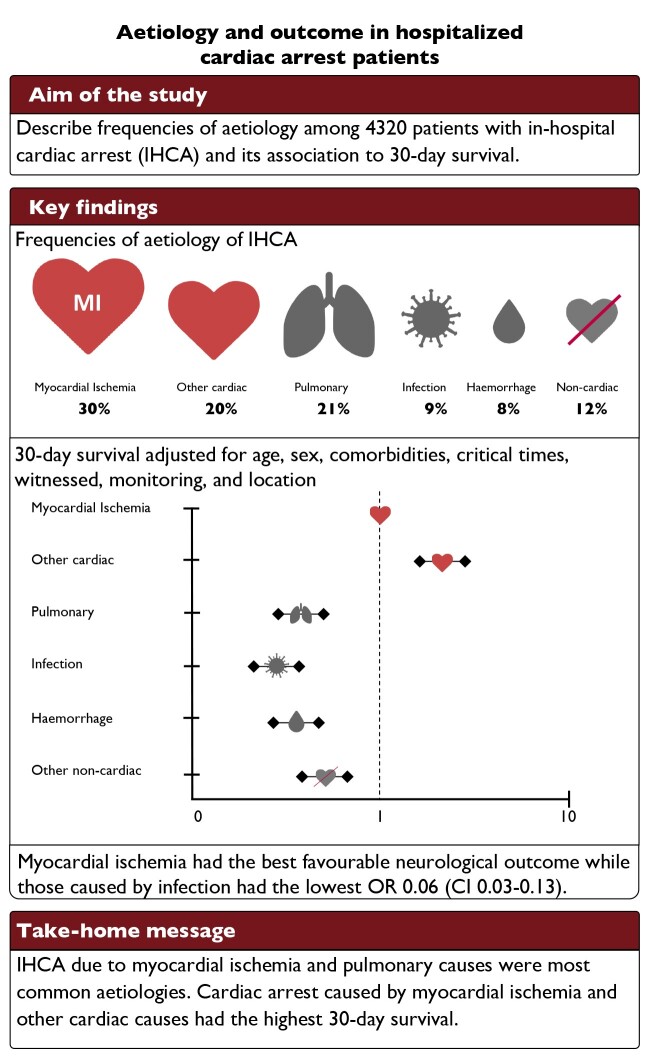

## Introduction

In-hospital cardiac arrest (IHCA) is a major health concern associated with high mortality and neurologic disabilities among survivors. The incidence of IHCA varies between 1.5 and 7 per 1000 hospital admissions depending on country, with a somewhat higher reported incidence in the US compared to Europe.^1–4^ The 30-day survival among reported IHCA patients varies between 15% and 34%.^3–8^

To reduce the incidence and improve outcome for patients suffering from IHCA, early recognition of clinical deterioration as well as recognition and treatment of the aetiology of the cardiac arrest is important.^9,10^ IHCA is often preceded by hours or days of clinical deterioration.^10^ Identification of the aetiology by the hospital's rapid response team (RRT) is often challenging. In the majority of cases, recognition of the aetiology is based on knowledge of the most common aetiologies of cardiac arrest and symptoms presented before the arrest.^11^ However, due to the emergency of the situation, information of the specific patient in medical charts and reported by staff is often difficult to fully grasp.

Improved identification of aetiology of IHCA is important as it affects both the potential to use specific treatments and outcome.^9,10^ Previous studies have shown acute myocardial infarction to be the most common aetiology of IHCA.^9,10,12^ Approximately 50% of the aetiology in patients with IHCA have in previous studies been of cardiac cause and 50% of non-cardiac cause.^11–13^ Most cardiac arrest studies are in the setting of out-of-hospital cardiac arrest, focusing on patients with a presumed cardiac cause with an initial shockable rhythm.^14–16^ A presumed cardiac cause is shown beneficial compared to non-cardiac causes as it is more prone to have an initial shockable rhythm for which defibrillation is an effective initial treatment.^2,4,12,17^ Patients presenting with an initial non-shockable rhythm are less well studied both in the in-hospital and out-of-hospital setting. These patients are associated with a higher mortality, and it is important to early assess potentially reversible causes to improve outcome in this group.^9^

Since 2005, the Swedish Registry of Cardiopulmonary Resuscitation (SRCR) for IHCA have collected data in a structured manner regarding aetiologies of the arrest, resuscitation factors, and outcomes in this patient group. In 2018, the aetiology categories for IHCA were expanded from eight to 22 categories to better understand the underlying factors, allowing a more detailed analysis of the causes behind IHCA and the outcomes in these patients. The aim of the present study is to study the proportion of aetiologies (main categories and detailed) of IHCA and to study their association to return of spontaneous circulation (ROSC), 30-day survival, and favourable neurologic outcome at discharge.

## Method

### Study design and ethics

This is an observational study based on data from the SRCR as well as the Cause of Death Registry and the Swedish Inpatient Registry from the National Board of Health and Welfare. The study was approved by the Swedish Ethical Review Authority, (Dnr 2021-00260).

The SRCR is a national quality registry with the primary aim to improve knowledge and quality of care among patients who suffer from cardiac arrest in Sweden. The registration of IHCA started in 2005 and since then the number of participating hospitals has increased. From 2018, the registry includes all 73 emergency hospitals in Sweden.^18^ In 2018, the SRCR expanded the number of categories for recording the aetiology of IHCA, from eight to 22 categories. This change enabled a more detailed description of the aetiology of IHCA. Data in the registry are collected according to the Utstein template however the Utstein template does not specify how aetiology of IHCA should be reported.^19,20^ Patients with a do not resuscitate order are not included in the SRCR.

### National Board of Health and Welfare

The Swedish Inpatient Registry includes the primary and secondary discharge diagnoses based on ICD10 for inpatient care throughout Sweden.^21^ The Inpatient Registry includes hospitalizations since 1987 and has been validated.^22^ Since 1991, all deaths in Sweden are reported to the Cause of Death Registry and approximately 92 000 deaths are reported yearly.^23^

### Patient inclusion and exclusion criteria

The study included patients ≥18 years of age who suffered from IHCA, received cardiopulmonary resuscitation (CPR), and were registered in the SRCR between 18th of April 2018 and 31st December 2020. Patients with ongoing CPR upon admission to the hospital were excluded. In cases where the patient experienced cardiac arrest more than once, only the first cardiac arrest was included.

### Registration of IHCA aetiology

**Figure 1:**
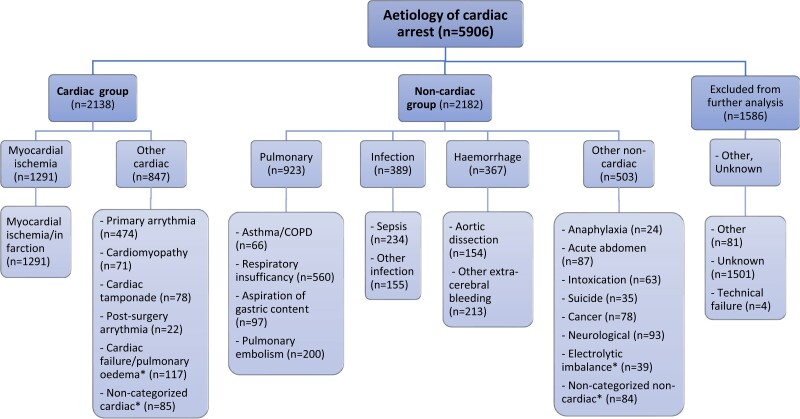
Categorisation and frequencies of aetiologies among patients who experienced IHCA during the study period. *Aetiology categories added by the authors.

Before April 2018, the aetiology of cardiac arrest was recorded in eight categories in the SRCR. In April 2018, the number of categories expanded to 22 categories. The instructions to the reporting nurse or physician regarding aetiologies to the cardiac arrest is to register the ‘primary triggering cause of the cardiac arrest’. For each patient with IHCA, only one aetiology category can be chosen. *Figure 1* presents the 22 aetiologies reported to the SRCR. In this study, the 22 categories were grouped into six main categories: myocardial ischemia, other cardiac causes, pulmonary causes, infection, haemorrhage, and other non-cardiac causes. These six main categories were then further categorized in cardiac and non-cardiac group (*Figure 1*). Supplementary material online, *Figure S1* presents the SRCR's explanation of each of the 22 categories.

**Figure 2:**
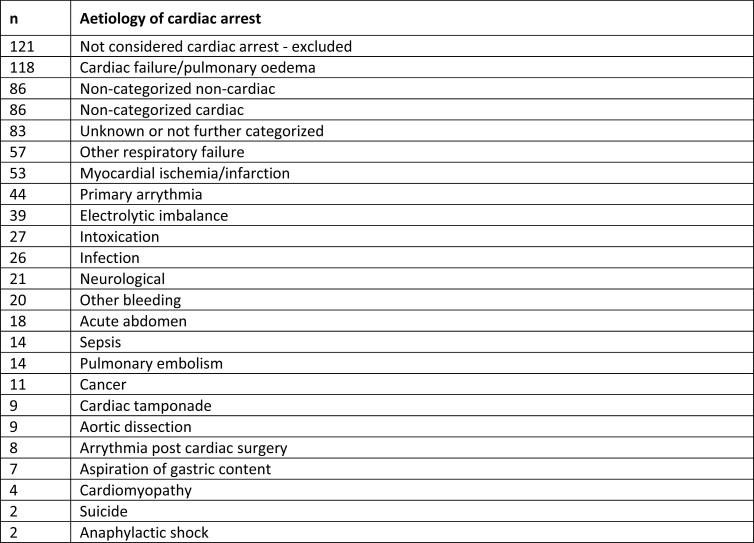
Aetiologies primarily categorized as “other” but with complimentary information from the SRCR categorized in the 26 aetiology categories.

Of the patients who had the IHCA aetiology registered as ‘other’ (*n* = 879), 856 (97.4%) could be categorized into one of the 22 standard categories by reading free text added by the reporting nurse or physician. Four additional aetiology categories were added: cardiac failure/pulmonary oedema, non-categorized cardiac cause (for example aortic stenosis or procedure related), electrolyte imbalance, and non-categorized non-cardiac cause (for example liver failure, ketoacidosis, hypovolemia). Patients who could not be categorized were excluded from further analyses. *Figure 1* and *Figure 2* show a summary of the categorization of cardiac arrest registered as ‘other’.

### Data collection and linkage

As described in previous studies, the registration in the SRCR occurs in two steps.^24^ The first part of the registration is performed by a nurse or physician who were present at the scene of the cardiac arrest. This part contains information regarding treatments given during the resuscitation, place of the cardiac arrest, critical delay times, initial rhythm, if the patient was monitored at the time of the arrest, and if the patient received ROSC. As these variables most often are collected at the time of the acute event, situations may occur where there are limitations in the possibility to report some of the variables.

After death or 30-days, a registered nurse or physician affiliated with the SRCR fills in the second part of the registration based on information in patient's medical chart. This part contains information of probable aetiology of the cardiac arrest, clinical condition hours prior to the cardiac arrest, treatment after resuscitation, 30-day survival, Cerebral Performance Category (CPC) score at hospital discharge, and previous medical history.

Patients who suffered from IHCA during the study period were identified in the SRCR. By using the 12-digit personal identification number, unique for all Swedish citizens, data from the Cause of Death Registry and the Swedish Inpatient Registry from the National Board of Health and Welfare were merged seamless.

### Outcomes

We present frequency of IHCA aetiologies, ROSC, 30-day survival, and favourable neurological outcome at hospital discharge. ROSC was defined as sustained ROSC for a minimum of 20 min. Favourable neurological outcome was considered as having a CPC score of 1–2 at hospital discharge. CPC-score is a five-point scale ranging from 1–5, CPC 1 representing good cerebral performance and CPC 5 representing brain death.

### Data analysis and statistical methods

Baseline characteristics were reported using median with appropriate dispersions measures. The two aetiology groups, cardiac and non-cardiac group, were compared using Chi square test for dichotomous variables and Wilcoxon two-sample test for continuous variables. When the aetiologies were categorized in the six main categories, analysis were performed using multiple logistic regression analysis. A *P* < 0.05 was considered significant.

The association between aetiology and outcome was analysed with multivariable logistic regression model, described using odds ratio (OR) with 95% confidence interval and presented as forest plots. In the adjusted models, missing data were imputed using multiple imputation by chain equations (mice). Regression analyses were performed on 10 imputed datasets and the results were pooled using Rubin's rule. Data was assumed to be missing at random. The same models with complete cases analysis are presented in supplement. Cardiac arrest due to myocardial ischemia was set as reference. Three different models are presented. Model 1 presents the unadjusted 30-day survival. Model 2 presents adjusted 30-day survival, confounders adjusted for include sex, age, and comorbidity. Model 3 presents adjusted 30-day survival and include model 2 and intermediate variables known to affect outcome including if CPR was started within 1 min, if the patient was monitored or witnessed at the time of the arrest, and the location of the cardiac arrest. Initial rhythm was not adjusted for, as it is closely related to aetiology and will increase net bias by overadjustment of the model. The same models were used for ROSC and favourable neurological outcome CPC 1–2 at hospital discharge. The statistical analysis was performed using R 4.1.3.

## Results

### Baseline characteristics

A total of 5906 patients suffered from IHCA and were included in the study of whose aetiologies were registered for 4324 patients (73.2%). The median age of all patients with a registered aetiology were 74 years, and 2729/4324 (63.1%) were men. The proportion of male patients in the cardiac group was higher compared to the non-cardiac group (68% vs. 58.2%, *P* < 0.001). Patients with cardiac arrest due to a non-cardiac cause had significantly more comorbidities compared to cardiac arrest of a cardiac cause (*Tables 1* and *2*). In the six main aetiology categories cardiac arrest due to other non-cardiac causes were the youngest group with a median age of 72 years (*Table 3*). Apart from hypertension, cancer was the second most common comorbidity in all four aetiology categories in the non-cardiac group. Cardiac arrest due to pulmonary cause had the highest proportion of asthma and Chronic Obstructive Pulmonary Disease. Cardiac arrest due to infection had the highest proportion of diabetes (*Table 3*). Information regarding initial cardiac rhythm, time, and treatment of IHCA of the six main aetiology categories is presented in (*Table 4*). Basic characteristics among patients in all 26 aetiologies are presented in Supplementary material online, *Tables S1:1–S1:4* and *S2:1–S2:4*.

**Table 1:** Basic characteristics presented in the cardiac and non-cardiac groups

	Cardiac cause (%)	Non cardiac cause (%)	*P*-value	SMD	Missing (%)
*n*	2138	2182			
Sex (Women)	689 (32.2)	906 (41.5)	<0.001	0.194	0.0
Age [median (IQR)]	74 [66, 81]	73 [65, 80]	0.020	0.113	0.0
**Comorbidity**					
Cancer	700 (32.7)	886 (40.6)	<0.001	0.164	0.0
Diabetes	641 (30.0)	642 (29.4)	0.712	0.012	0.0
Renal failure	374 (17.5)	489 (22.4)	<0.001	0.123	0.0
Heart failure	733 (34.3)	636 (29.1)	<0.001	0.111	0.0
Myocardial infarction	1185 (55.4)	528 (24.2)	<0.001	0.673	0.0
Asthma/COPD	300 (14.0)	479 (22.0)	<0.001	0.207	0.0
Addiction	253 (11.8)	411 (18.8)	<0.001	0.195	0.0
Psychiatric comorbidity	154 (7.2)	280 (12.8)	<0.001	0.188	0.0
Hypertension	1357 (63.5)	1381 (63.3)	0.927	0.004	0.0
**Location**			<0.001	1.048	0.0
Emergency department	274 (12.8)	328 (15.0)			
Catheterization lab	478 (22.4)	17 (0.8)			
Other	44 (2.1)	38 (1.7)			
Cardiac care unit (CCU)	482 (22.5)	116 (5.3)			
Intermediate care unit (IMCU)	23 (1.1)	85 (3.9)			
Intensive care unit (ICU)	144 (6.7)	228 (10.4)			
Clinic, laboratory, radiology department	67 (3.1)	135 (6.2)			
Operation room (OR)	26 (1.2)	57 (2.6)			
General ward	600 (28.1)	1178 (54.0)			

**Table 2:** Information of initial cardiac rhythm, circumstances, time, and treatment of IHCA presented in the cardiac and non-cardiac groups

	Cardiac cause (%)	Non cardiac cause (%)	*P*-value	SMD	Missing (%)
*n*	2138	2182			
**Initial cardiac rhythm**			<0.001	0.879	18.7
Asystole	596 (31.0)	794 (46.5)			
Pulseless electrical activity (PEA)	472 (24.6)	761 (44.5)			
Ventricular fibrillation (VF)	586 (30.5)	97 (5.7)			
Pulseless ventricular tachycardia (VT)	268 (13.9)	57 (3.3)			
Witnessed	1905 (89.2)	1701 (78.5)	<0.001	0.295	0.6
ECG monitored	1641 (77.6)	953 (44.6)	<0.001	0.719	1.5
**Event times**					
Recognition—call ≤1min	1408 (84.7)	1408 (78.9)	<0.001	0.149	19.2
Recognition—CPR ≤1min	1780 (93.8)	1835 (89.8)	<0.001	0.145	8.8
Recognition—def ≤3min	754 (79.4)	127 (38.6)	<0.001	0.911	72.4
**Treatment at the scene of the arrest**					
CPR started before arrival of RRT	1625 (91.2)	1761 (92.1)	0.404	0.029	14.0
Chest compression	1539 (98.3)	1703 (99.4)	0.005	0.102	23.6
Defibrillation performed	1025 (49.0)	358 (16.8)	<0.001	0.728	3.0
Intubation	831 (39.8)	1291 (60.8)	<0.001	0.468	3.4
Epinephrine	1152 (55.0)	1629 (76.3)	<0.001	0.491	2.7
**Post cardiac arrest treatment**					
PCI	509 (41.5)	10 (1.0)	<0.001	1.062	48.6
ICD	113 (9.2)	2 (0.2)	<0.001	0.418	48.6
Pacemaker during hospital stay	268 (21.8)	19 (2.0)	<0.001	0.629	48.6
Coronary angiography	571 (46.5)	29 (3.0)	<0.001	1.066	48.6
CABG during hospital stay	25 (2.0)	1 (0.1)	<0.001	0.183	48.6

**Table 3:** Basic characteristics presented in the six main aetiology categories

Cardiac or non-cardiac group	Cardiac group		Non-cardiac group				Missing (%)
6 main aetiology categories	Myocardial ischemia (%)	Other cardiac cause (%)	Pulmonary causes (%)	Infection (%)	Haemorrhage (%)	Other non-cardiac (%)	
*N*	1291 (29.9)	847 (19.8)	923 (21.4)	389 (9)	367 (8.5)	503 (11.6)	
Sex (Women)	406 (31.4)	283 (33.4)	394 (42.7)	139 (35.7)	146 (39.8)	227 (45.1)	0.0
Age [median (IQR)]	74 [65, 81]	74 [66, 81]	74 [65, 80]	74 [66, 82]	75 [66, 80]	72 [59, 79]	0.0
**Comorbidity**							
Cancer	416 (32.2)	284 (33.5)	386 (41.8)	150 (38.6)	142 (38.7)	208 (41.4)	0.0
Diabetes	376 (29.1)	265 (31.3)	265 (28.7)	146 (37.5)	85 (23.2)	146 (29.0)	0.0
Renal failure	180 (13.9)	194 (22.9)	183 (19.8)	116 (29.8)	76 (20.7)	114 (22.7)	0.0
Heart failure	345 (26.7)	388 (45.8)	305 (33.0)	151 (38.8)	78 (21.3)	102 (20.3)	0.0
Myocardial infarction	854 (66.2)	331 (39.1)	225 (24.4)	115 (29.6)	90 (24.5)	98 (19.5)	0.0
Asthma/COPD	158 (12.2)	142 (16.8)	256 (27.7)	84 (21.6)	51 (13.9)	88 (17.5)	0.0
Addiction	139 (10.8)	114 (13.5)	164 (17.8)	66 (17.0)	58 (15.8)	123 (24.5)	0.0
Psychiatric comorbidity	80 (6.2)	74 (8.7)	112 (12.1)	51 (13.1)	31 (8.4)	86 (17.1)	0.0
Hypertension	784 (60.7)	573 (67.7)	589 (63.8)	267 (68.6)	235 (64.0)	290 (57.7)	0.0
**Location**							0.0
Emergency department	181 (14.0)	93 (11.0)	133 (14.4)	34 (8.7)	89 (24.3)	72 (14.3)	
Catherization lab	410 (31.8)	68 (8.0)	6 (0.7)	0 (0.0)	7 (1.9)	4 (0.8)	
Other	24 (1.9)	20 (2.4)	16 (1.7)	2 (0.5)	4 (1.1)	16 (3.2)	
Cardiac care unit (CCU)	287 (22.2)	195 (23.0)	63 (6.8)	13 (3.3)	17 (4.6)	23 (4.6)	
Intermediate care unit (IMCU)	10 (0.8)	13 (1.5)	39 (4.2)	25 (6.4)	10 (2.7)	11 (2.2)	
Intensive care unit (ICU)	64 (5.0)	80 (9.4)	87 (9.4)	65 (16.7)	27 (7.4)	49 (9.7)	
Clinic, laboratory, radiology department	30 (2.3)	37 (4.4)	36 (3.9)	16 (4.1)	37 (10.1)	37 (10.1)	
Operation room (OR)	6 (0.5)	20 (2.4)	16 (1.7)	8 (2.1)	13 (3.5)	20 (4.0)	
General ward	279 (21.6)	321 (37.9)	527 (57.1)	226 (58.1)	163 (44.4)	262 (52.1)	

**Table 4:** Information of initial cardiac rhythm, circumstances, time, and treatment of IHCA presented in the six main aetiology categories

Cardiac or non-cardiac group	Cardiac group		Non-cardiac group				
6 main aetiology categories	Myocardial ischemia (%)	Other cardiac cause (%)	Pulmonary causes (%)	Infection (%)	Haemorrhage (%)	Other non-cardiac (%)	Missing (%)
*n*	1291 (29.9)	847 (19.8)	923 (21.4)	389 (9)	367 (8.5)	503 (11.6)	
**Initial cardiac rhythm**							18.7
Asystole	287 (24.6)	309 (40.9)	323 (45.5)	159 (50.8)	120 (41.4)	192 (48.5)	
Pulseless electrical activity (PEA)	300 (25.7)	172 (22.8)	343 (48.3)	122 (39.0)	152 (52.4)	144 (36.4)	
Ventricular fibrillation (VF)	443 (38.0)	143 (18.9)	23 (3.2)	24 (7.7)	14 (4.8)	36 (9.1)	
Pulseless ventricular tachycardia (VT)	137 (11.7)	131 (17.4)	21 (3.0)	8 (2.6)	4 (1.4)	24 (6.1)	
Witnessed	1170 (90.8)	735 (86.9)	734 (80.0)	290 (75.5)	294 (80.8)	383 (76.4)	0.6
ECG monitored	1041 (81.6)	600 (71.4)	387 (43.0)	178 (46.4)	174 (48.5)	214 (43.2)	1.5
**Event times**							
Recognition—call ≤1min	835 (85.6)	573 (83.4)	582 (77.1)	249 (80.6)	262 (84.2)	315 (77.0)	19.2
Recognition—CPR ≤1min	1068 (94.0)	712 (93.4)	776 (90.1)	335 (90.8)	316 (91.3)	408 (87.4)	8.8
Recognition—def ≤3min	526 (80.6)	228 (76.8)	43 (37.1)	25 (37.3)	17 (32.1)	42 (45.2)	72.4
**Treatment at the scene of the arrest**							
CPR started before arrival of RRT	951 (90.2)	674 (92.7)	753 (92.4)	310 (94.5)	299 (90.6)	399 (90.7)	14.0
Chest compression	894 (97.8)	645 (99.1)	728 (99.5)	300 (99.7)	288 (99.0)	387 (99.5)	23.6
Defibrillation performed	694 (54.8)	331 (40.0)	117 (13.0)	76 (20.2)	59 (16.3)	106 (21.6)	3.0
Intubation	547 (43.3)	284 (34.5)	556 (62.1)	230 (60.4)	236 (66.3)	269 (55.0)	3.4
Epinephrine	734 (57.9)	418 (50.4)	707 (78.3)	311 (81.0)	284 (78.7)	327 (67.3)	2.7
**Post cardiac arrest treatment**							
PCI	492 (59.9)	54 (9.0)	4 (0.7)	1 (0.5)	2 (1.4)	6 (1.9)	48.6
ICD	44 (5.4)	86 (14.3)	2 (0.4)	0 (0.0)	0 (0.0)	3 (1.0)	48.6
Pacemaker during hospital stay	78 (9.5)	236 (39.3)	7 (1.3)	6 (3.1)	1 (0.7)	15 (4.8)	48.6
Coronary angiography	493 (60.0)	137 (22.8)	18 (3.4)	2 (1.0)	3 (2.1)	25 (8.0)	48.6
CABG during hospital stay	24 (2.9)	4 (0.7)	2 (0.4)	0 (0.0)	0 (0.0)	0 (0.0)	48.6

### Frequency of aetiologies

**Figure 3:**
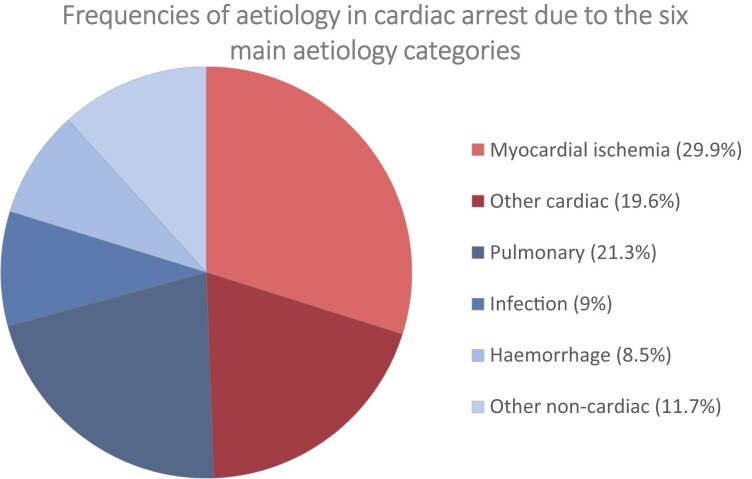
Frequency distribution of the six main aetiology categories.

The distribution between the cardiac group and the non-cardiac group were 49.4% vs. 50.6% (*Table 1*). The most common aetiology was myocardial ischemia (29.9%) followed by pulmonary causes (21.3%), other cardiac causes (19.6%), other non-cardiac causes (11.7%), infection (9%), and haemorrhage (8.5%) (*Figure 3*). Supplementary material online, *Figures S2* and *S3* present the frequencies of all 26 aetiologies divided into cardiac and non-cardiac causes.

### Thirty-day survival

The cardiac group had significantly higher 30-day survival (48.4% vs. 18.7%, *P* < 0.001) compared to the non-cardiac group (*Table 5*). In the six main aetiology categories, cardiac arrest with an aetiology of other cardiac causes had the highest 30-day survival (53.5%) followed by cardiac arrest due to myocardial ischemia (43.8%) (*Table 6*). Lowest crude 30-day survival was seen in cardiac arrest due to haemorrhage (14.7%). Overall, 30-day survival in all 26 aetiology categories varied between (2.6% and 75%) and are presented in Supplementary material online, *Tables S3:1–S3:4*.

**Table 5:** Outcomes presented in the cardiac and non-cardiac group

	Cardiac cause (%)	Non cardiac cause (%)	*P*-value	Missing (%)
*n*	2138	2182		
ROSC (%)	1361 (64.4)	1108 (51.6)	<0.001	1.8
30-day survival (%)	1018 (47.6)	472 (21.6)	<0.001	0.0
**CPC (%)**			<0.001	72.7
CPC 1–2	900 (89.5)	321 (73.0)		
CPC 3–5	42 (4.2)	88 (20.0)		
Unknown CPC	64 (6.4)	31 (7.0)		

**Table 6:** Outcomes presented in the six main aetiology categories

Cardiac or non-cardiac group	Cardiac group		Non-cardiac group				*P*
6 main aetiology categories	Myocardial ischemia (%)	Other cardiac cause (%)	Pulmonary causes (%)	Infection (%)	Haemorrhage (%)	Other non-cardiac (%)	
*n*	1291 (29.9)	847 (19.6)	923 (21.3)	389 (9)	367 (8.5)	503 (11.7)	
ROSC (%)	784 (61.5)	577 (68.9)	503 (55.5)	184 (47.5)	130 (35.8)	291 (59.0)	<0.001
30-day survival (%)	565 (43.8)	453 (53.5)	203 (22.0)	63 (16.2)	54 (14.7)	152 (30.2)	<0.001
**CPC (%)**							<0.001
CPC 1–2	515 (91.2)	385 (87.3)	138 (72.3)	32 (54.2)	44 (88.0)	107 (76.4)	
CPC 3–5	18 (3.2)	24 (5.4)	43 (22.5)	18 (30.5)	4 (8.0)	23 (16.4)	
Unknown CPC	32 (5.7)	32 (7.3)	10 (5.2)	9 (15.3)	2 (4.0)	10 (7.1)	

**Figure 4:**
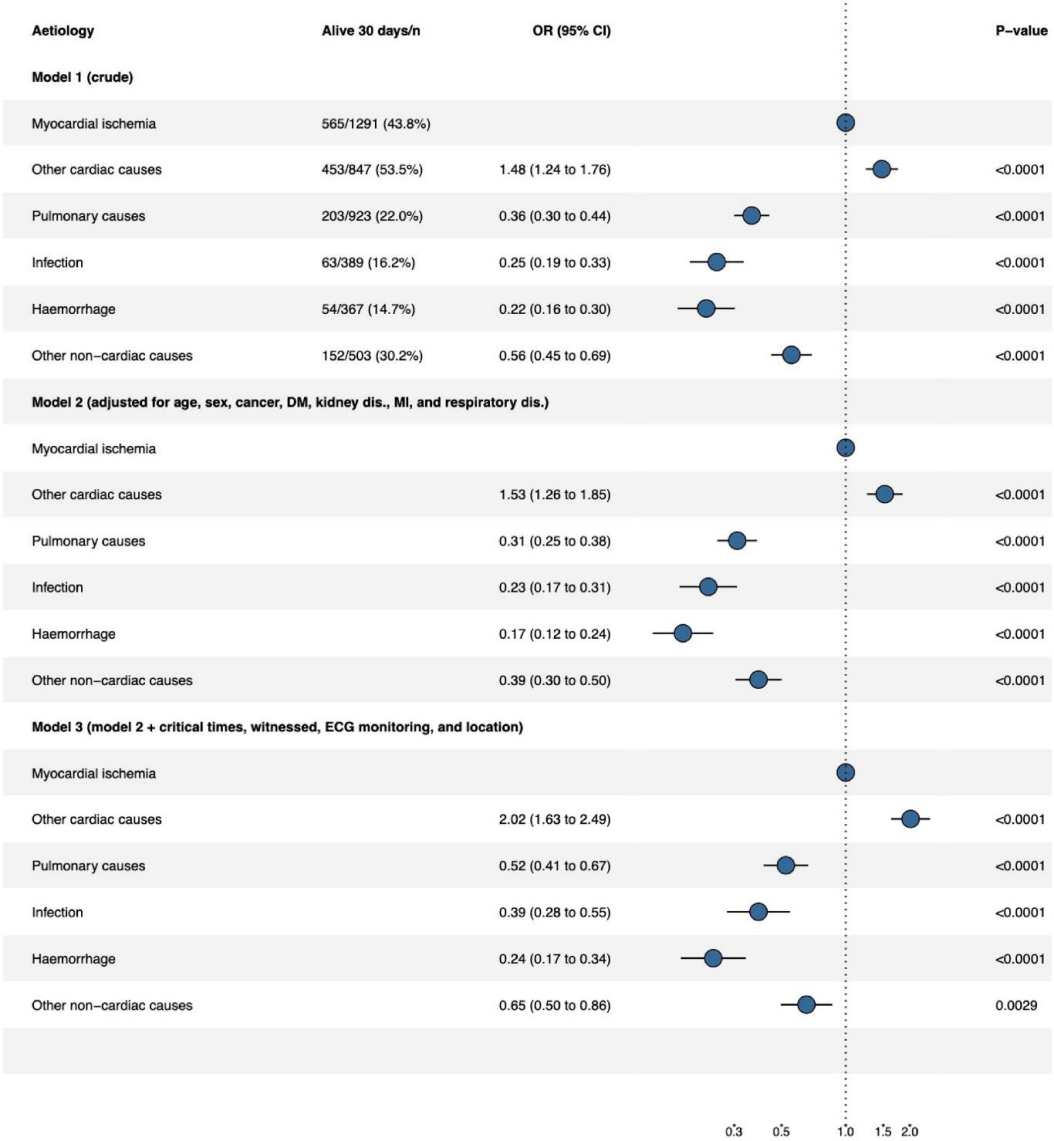
Forrest plot of 30-day survival. Model 1, unadjusted 30-day survival. Model 2, adjusted for confounders. Model 3, adjusted for confounders and intermediates.

*Figure 4*, model 1 presents the OR of unadjusted 30-day survival among patients who suffer from IHCA. All four aetiologies in the non-cardiac group had a lower probability of 30-day survival compared to the two aetiologies in the cardiac group. The OR for 30-day survival compared to myocardial ischemia were, for other cardiac causes OR 1.48 (CI 1.24–1.76), pulmonary causes OR 0.36 (CI 0.3–0.44), infection OR 0.25 (CI 0.18–0.33), haemorrhage OR 0.22 (CI 0.16–0.3), and other non-cardiac causes OR 0.56 (CI 0.45–0.69). When adjusting for confounders as well as when adjusting for confounders and intermediates, the lower 30-day survival among aetiologies in the non-cardiac group (pulmonary, infection, haemorrhage, and other non-cardiac causes) remained (*Figure 4*, model 2 and 3). Complete case analysis is presented in Supplementary material online, *Figure S4*.

### Return of spontaneous circulation

Cardiac arrest due to cardiac cause had significantly higher ROSC (65.3% vs. 49.9%, *P* < 0.001), compared to cardiac arrest due to non-cardiac cause (*Table 5*). Among the six main aetiology categories, ROSC were highest among patients who suffered from cardiac arrest due to other cardiac causes (68.9%). Cardiac arrest due to haemorrhage had the lowest ROSC (35.8%) (*Table 6*). Overall, in the detailed aetiology categories, ROSC were ranging between 20.4% and 95.5% (cardiac arrest due to aortic dissection having the lowest and cardiac arrest due to anaphylactic shock having the highest proportion of ROSC) (see Supplementary material online, *Tables S3:1–S3:4*).

**Figure 5:**
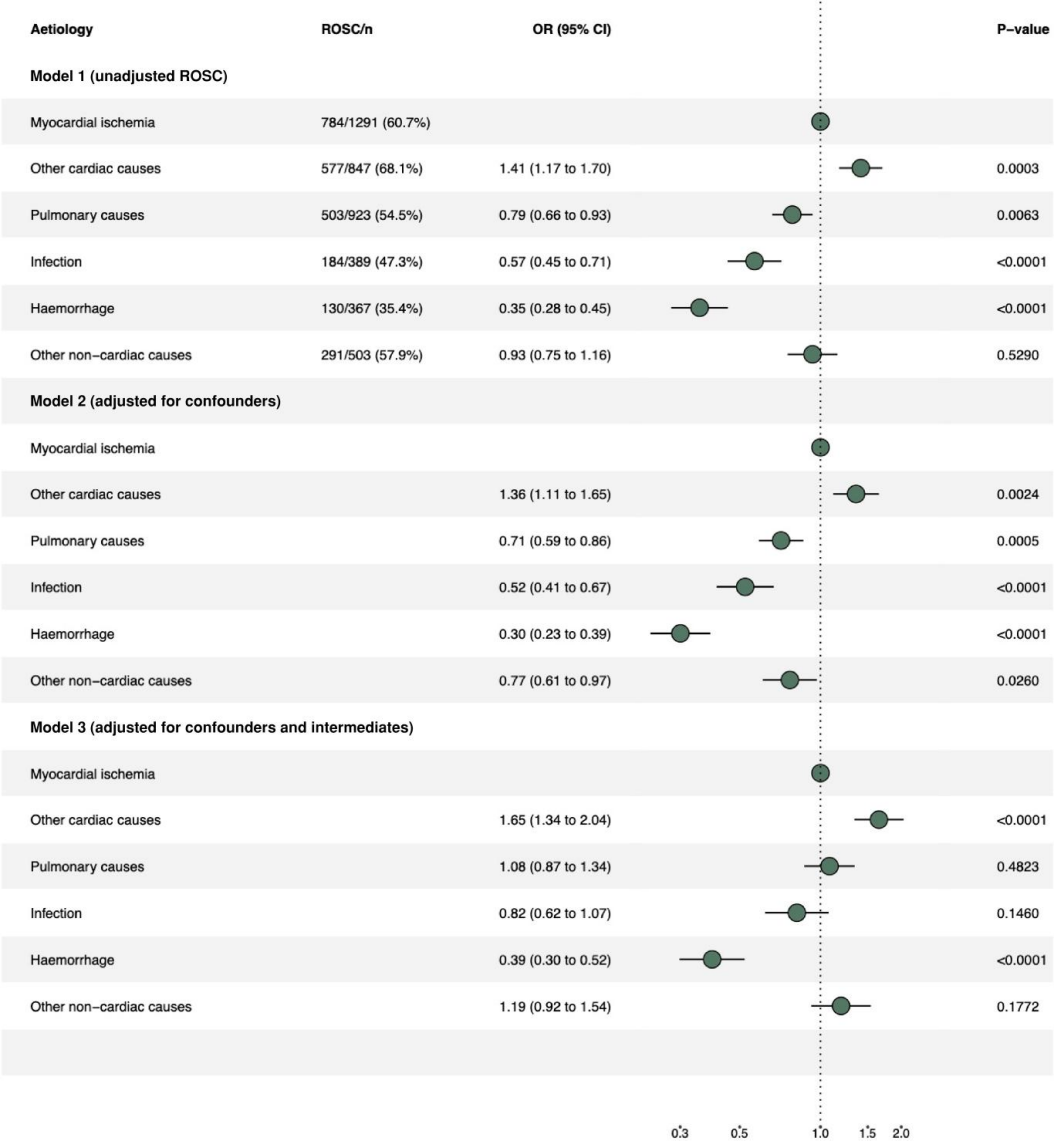
Forrest plot of ROSC. Model 1, unadjusted ROSC. Model 2, adjusted for confounders. Model 3, adjusted for confounders and intermediates.

When adjusting for confounders, the proportion of ROSC in all four aetiologies in the non-cardiac group had a lower OR of ROSC compared to myocardial ischemia (*Figure 5*, model 2). The lower OR of ROSC seen among cardiac arrests due to infection, pulmonary cause, and other non-cardiac causes were no longer significant when adjusting for both confounders and intermediates (*Figure 5*, model 3). However, cardiac arrest due to haemorrhage still had a lower OR for ROSC compared to cardiac arrest due to myocardial ischemia. Complete case analysis is presented in Supplementary material online, *Figure S5*.

### Favourable neurological outcome, Cerebral Performance Category score

The cardiac group had significantly higher favourable neurological outcome (89.8% vs. 72.8%, *P* < 0.001) compared to the non-cardiac group (*Table 5*). The highest proportion of favourable neurological outcome at hospital discharge among survivors were seen among patients with an aetiology of myocardial ischemia, with 91.2% of survivors having a CPC-score of 1–2 (*Table 6*). Among patients with pulmonary cause, 72.3% had favourable neurological outcome at discharge. The lowest proportion of favourable neurological outcome was seen among patients who suffered from cardiac arrest due to infection (54.2%). In the detailed aetiology categories, CPC-score of 1–2 at hospital discharge, were ranging between 42.9 and 100% (cardiac arrest due to stroke having the lowest and cardiac arrest due to aortic dissection having the highest of CPC-score 1–2) (Supplementary material online, *Tables S3:1–S3:4*).

**Figure 6:**
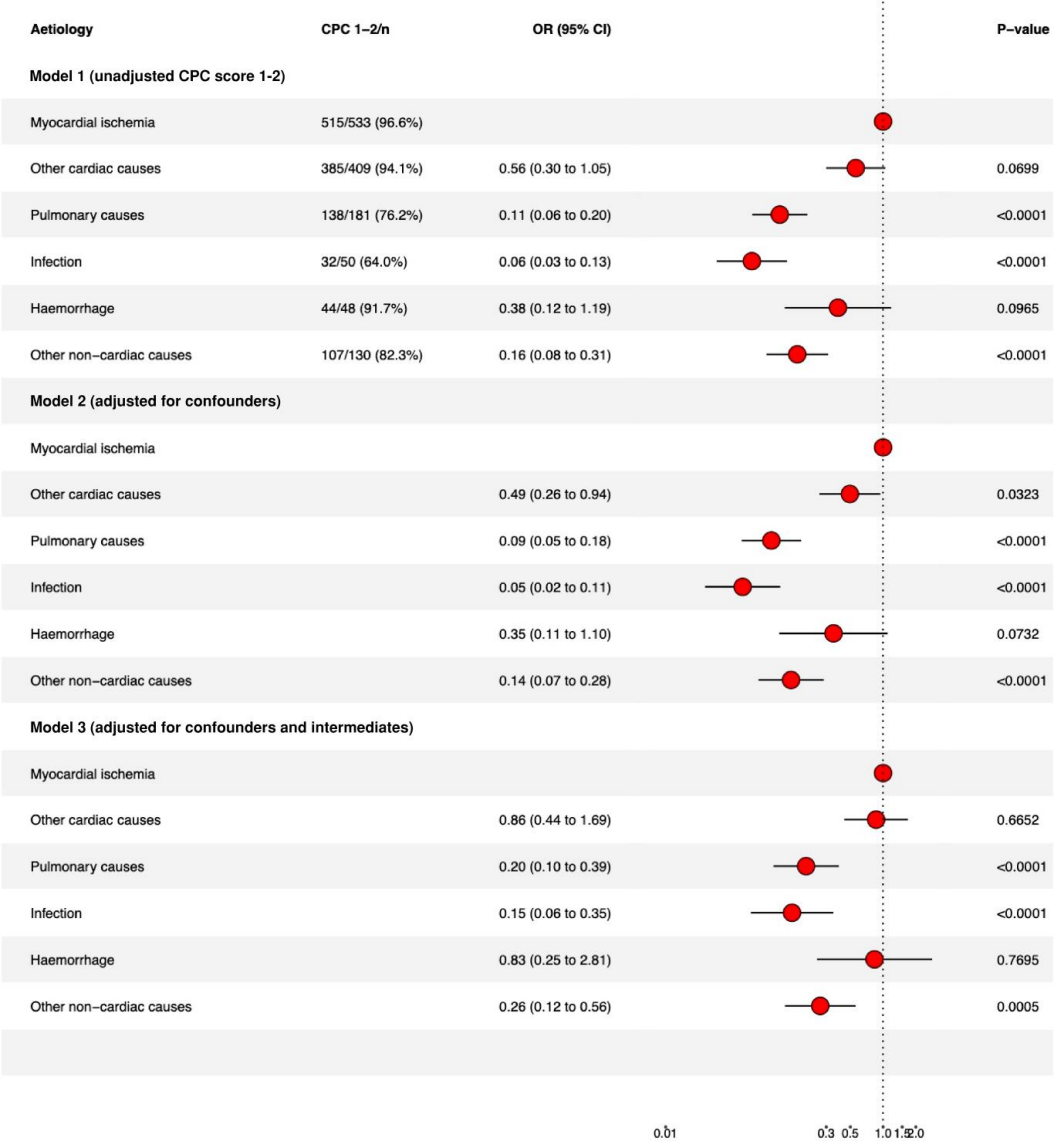
Forrest plot of favourable neurological outcome among survivors (CPC-score 1–2). Model 1, unadjusted CPC-score 1–2. Model 2, adjusted for confounders. Model 3, adjusted for confounders and intermediates.

In the adjusted analysis, the lower OR of favourable neurological outcome among cardiac arrest due to infection, pulmonary, and other non-cardiac causes remained when adjusting for confounders as well as when adjusting for confounders and intermediates (*Figure 6*). Complete case analysis is presented in Supplementary material online, *Figure S6*.

## Discussion

Identifying aetiology may enable prevention of the cardiac arrest and has implication on treatment and outcome. Apart from correctly performed CPR, urgent work must be done to treat the underlying pathophysiology leading up to the cardiac arrest. Many cardiac arrests are considered preventable in retrospective reviews. To prevent cardiac arrest and improve survival, it is important to have a system both for identifying patients at risk of deteriorating to cardiac arrest and to have a RRT with knowledge of appropriate interventional responses. Studies have shown that the benefit of recognizing the aetiology of cardiac arrest is more pronounced in non-cardiac causes and non-shockable rhythms which may correspond to possible reversible aetiologies.^9,17^ Cardiac arrest due to non-cardiac causes is a more heterogenic group and treatment of the underlying cause is more diverse and not as obvious as for myocardial ischemia where revascularization is gold standard. For non-cardiac causes, treatment such as thrombolysis may be vital for a patient with cardiac arrest due to pulmonary embolism but devastating for a patient with dissecting aortic aneurysm or intracerebral bleeding. Also, some aetiologies have especially low 30-day survival and among other aetiologies patients have an increased risk of surviving with neurological impairment. This is important knowledge for clinicians both in the treatment of the patient peri and post cardiac arrest, but also in the communication with relatives. These findings are important and support that there is a need for a universal categorization of the aetiology of IHCA.

The main findings of this nationwide study of IHCA with more granular data on aetiologies compared to previous studies were that cardiac arrest due to myocardial ischemia and pulmonary cause were the two most common causes with considerable differences in ROSC, 30-day survival, and neurologic outcomes. Patients whose cardiac arrest was caused by myocardial ischemia and other cardiac causes had the highest proportion of ROSC and 30-day survival. When comparing outcomes among all registered aetiologies, there was a wide range in the proportion of achieving ROSC (20.4–95.5%), 30-day survival (2.6–75%), and CPC 1–2 at discharge (42.9–100%). In the comparison between the cardiac and non-cardiac group, the main finding was that the differences in outcome between the two groups are most pronounced in the 30-day survival and functional neurologic outcome, not in ROSC.

The distribution of the six main aetiology categories in our study may be difficult to compare with other studies as there is not a uniform way to report aetiologies of IHCA and thus the frequency and aetiology categories differs between studies.^19,25^ In addition, the Utstein template for IHCA from 2019 does not specify the reporting of aetiology.^19^ Other studies have reported a prevalence of cardiac cause of 50–63% and pulmonary cause of 11–42%,^4,12,26–28^ thus, similar to our data. The prevalence of haemorrhage and infection as causes of IHCA is also similar.^4,9,12,13,25,29^ The study by Hessulf *et al.* is one of few studies from the SRCR that report frequencies of aetiologies of IHCA prior to the change from eight to 22 categories in 2018.^30^ In Hessulf's study 25% of the IHCA were due to primary arrhythmia, 34% due to myocardial ischemia, 11% due to respiratory failure, 7% due to hypotension and 4% due to acute pulmonary oedema. The remaining 19% did not have an aetiology registered and classified as missing. Compared to the study by Hessulf *et al.*, the non-cardiac group in our study was significantly larger and we were also able to classify the non-cardiac group more carefully.

Wallmuller *et al.* present a detailed description of aetiologies among IHCA and their relation to CPC-score at six-months.^12^ When comparing their data with our results (CPC 1–2 at hospital discharge), the proportion of favourable neurological outcome in our study were lower among patients with an aetiology of myocardial ischemia and most non-cardiac causes, but higher among other cardiac causes. However, these results are difficult to compare as the definition of aetiologies, the way data were retrieved, and the time from cardiac arrest to neurological evaluation were different.^12^

An important finding in our study was that patients with non-cardiac causes had worse outcomes, in particular 30-day survival and functional neurologic outcome, compared to patients with cardiac cause of IHCA. This is in accordance with previous studies.^4,12,26^ Cardiac arrests in the non-cardiac group more often occurred in general wards, in unmonitored patients, were less often witnessed, and more often had an initial non-shockable rhythm. The four aetiologies in the non-cardiac group also had a lower proportion of patients receiving CPR within one minute from recognition of the cardiac arrest compared to the cardiac group. A longer time of no circulation increases the hypoxic injury in all organs and especially the brain by each minute of prolonged delay. In addition, a delayed start of CPR increases the risk of conversion from a shockable to a non-shockable rhythm. All the above factors have in previous studies been associated with worse outcome.^30–33^ Patients with cardiac arrest due to infection, pulmonary, and other non-cardiac causes more often had symptoms of hypoxia one hour prior to cardiac arrest further increasing the risk of hypoxic organ damage and global ischemic injury post ROSC leading to multiorgan failure and a more severe post resuscitation syndrome. This may partly explain the more pronounced difference between ROSC and 30-day survival seen in the four aetiologies in the non-cardiac group as compared with the cardiac group.

We found some aetiologies with especially low 30-day survival including cardiac arrest due to aspiration, cancer, infection, sepsis, aortic dissection, cerebrovascular cause, and acute abdomen. An aetiology of aspiration and cancer has previously been shown to have particularly low survival.^24,30^ The treatment of the underlying cause in patients who suffer from cardiac arrest due to acute abdomen or aortic dissection, is immediate surgery. To perform surgery in a patient who recently was resuscitated after a cardiac arrest is high-risk performance and the low survival among these patients is thus not surprising. Sepsis is, regardless of cardiac arrest, associated with a high mortality. Cardiac arrest due to sepsis has multiple potential causes with multiple organ failure including circulatory failure, respiratory failure, vasoplegia, and metabolic derangement as potential causes.^34^ With early recognition of sepsis, acute abdomen, gastric impairment leading to aspiration, cardiac arrest due to these aetiologies may be preventable. Cardiac arrest due to cerebrovascular cause and cancer does not have a distinct treatment for the underlying cause and the survival for patients with these aetiologies will probably remain poor.

To be able to compare results and treatment of IHCA it is important to acknowledge the heterogenicity among cardiac arrests due to cardiac and non-cardiac causes. Thus, there is a need for harmonization when it comes to reporting aetiologies of IHCA.

### Strengths and limitation

The SRCR is a mandatory national register with high coverage, ensuring high quality data.

However, the study has several limitations. First, the aetiology of cardiac arrest is not always obvious, and the aetiology in a substantial proportion of the cardiac arrests will remain unknown unless autopsy is performed. In this study, 27% of the cardiac arrests had an unknown aetiology and this effects the internal validity. Second, the registration of data at the scene of the arrest such as critical delay times, treatment, and initial rhythm is challenging in acute situations and it is important to acknowledge that registry data have limitations in regard to recognition, collection, and encoding. However, this is true for the majority of registry studies especially when involving acute situations. This is considered as a random misclassification bias and would generally not contribute to systematic errors. Third, the cause of cardiac arrest may be multifactorial, in the SRCR only one aetiology can be chosen. Fourth, several of the aetiology categories pre-specified in the SRCR, such as infection and cancer are difficult to pathophysiologically explain as the triggering cause of the cardiac arrest. For the aetiologies infection and cancer, this rather indicates symptoms prior to the arrest rather than the final trigger of the cardiac arrest. The categorization of aetiologies of IHCA in SRCR is not standardised in the same way as in out-of-hospital cardiac arrest and further emphasises the need of a universal aetiology categorization.

## Conclusion

In this nationwide observational study of IHCA, the distribution between cardiac and non-cardiac causes were similar, but with a substantial difference in 30-day survival. Cardiac arrest caused by myocardial ischemia and other cardiac causes had the strongest associations with ROSC, 30-day survival and neurologic outcome compared to other categories. There is a need for a universal categorization of the aetiology of IHCA to be able to compare outcome, treatment, and intervention for IHCA in future studies.

## Supplementary Material

oead066_Supplementary_Data

## Data Availability

Data are available upon request from the author M.A.

## Lead author biography

Malin Albert is a doctoral student at the research group ‘Centre for Resuscitation Science’ at the Department of Clinical Science and Education, Södersjukhuset, Karolinska Institutet, Sweden. The title of the doctoral project is ‘aetiology, treatment, and prognostication of cardiac arrest’ and this article will be a part of the doctoral project. Malin Albert is also a last year resident physician at the department of anaesthesiology and intensive care at Södersjukhuset, Stockholm.
