# Supplementary material for: Aetiology and outcome in hospitalized cardiac arrest patients
Source: Eur Heart J Open. 2023 Jun 22;3(4):oead066. doi: 10.1093/ehjopen/oead066 (PMC10411044; doi:10.1093/ehjopen/oead066)

**Supplement**

| **Supplement table S1:1** Basic characteristics presented in the 26 aetiologies of cardiac arrest | | | | | | | |
| --- | --- | --- | --- | --- | --- | --- | --- |
| **Cardiac or non-cardiac group** | **Cardiac group** | | | | | | |
| **6 main aetiology categories** | **Myocardial ischemia** | **Other cardiac cause** | | | | | |
| **26 aetiology categories** | **Myocardial ischemia (%)** | **Cardiac tamponade (%)** | **Primary arrythmia (%)** | **Cardio-myopathy (%)** | **Arrythmia post cardiac surgery (%)** | **Cardiac failure (%)** | **Non-categorized cardiac (%)** |
| n | 1291 (29.9) | 78 (1.8) | 474 (11.0) | 71 (1.6) | 22 (0.5) | 117 (2.7) | 85 (2.0) |
| Sex (Women) | 406 (31.4) | 29 (37.2) | 158 (33.3) | 19 (26.8) | 5 (22.7) | 36 (30.8) | 36 (42.4) |
| Age (median [IQR]) | 74 [65, 81] | 75 [65, 81] | 74 [67, 81] | 73 [58, 79] | 73 [61, 77] | 76 [70, 83] | 71 [64, 77] |
| **Comorbidity** |  |  |  |  |  |  |  |
| Cancer | 416 (32.2) | 28 (35.9) | 156 (32.9) | 28 (39.4) | 7 (31.8) | 386 (41.8) | 23 (27.1) |
| Diabetes | 376 (29.1) | 14 (17.9) | 150 (31.6) | 23 (32.4) | 7 (31.8) | 265 (28.7) | 28 (32.9) |
| Renal failure | 180 (13.9) | 13 (16.7) | 92 (19.4) | 23 (32.4) | 4 (18.2) | 183 (19.8) | 17 (20.0) |
| Cardiac failure | 345 (26.7) | 22 (28.2) | 185 (39.0) | 54 (76.1) | 10 (45.5) | 305 (33.0) | 33 (38.8) |
| Myocardial infarction | 854 (66.2) | 38 (48.7) | 160 (33.8) | 31 (43.7) | 15 (68.2) | 225 (24.4) | 37 (43.5) |
| Asthma/COPD | 158 (12.2) | 14 (17.9) | 72 (15.2) | 10 (14.1) | 2 (9.1) | 256 (27.7) | 24 (28.2) |
| Addiction | 139 (10.8) | 10 (12.8) | 63 (13.3) | 12 (16.9) | 2 (9.1) | 164 (17.8) | 11 (12.9) |
| Psychiatric comorbidity | 80 (6.2) | 4 (5.1) | 41 (8.6) | 10 (14.1) | 1 (4.5) | 112 (12.1) | 5 (5.9) |
| Hypertension | 784 (60.7) | 58 (74.4) | 306 (64.6) | 45 (63.4) | 18 (81.8) | 589 (63.8) | 61 (71.8) |
| **Location** |  |  |  |  |  |  |  |
| Emergency department | 181 (14.0) | 9 (11.5) | 55 (11.6) | 10 (14.1) | 0 (0.0) | 13 (11.1) | 6 (7.1) |
| Catheterization lab | 410 (31.8) | 16 (20.5) | 17 (3.6) | 3 (4.2) | 1 (4.5) | 6 (5.1) | 25 (29.4) |
| Other | 24 (1.9) | 1 (1.3) | 12 (2.5) | 1 (1.4) | 0 (0.0) | 2 (1.7) | 4 (4.7) |
| Cardiac care unit (CCU) | 287 (22.2) | 22 (28.2) | 134 (28.3) | 11 (15.5) | 3 (13.6) | 14 (12.0) | 11 (12.9) |
| Intermediate care unit (IMCU) | 10 (0.8) | 1 (1.3) | 7 (1.5) | 3 (4.2) | 1 (4.5) | 0 (0.0) | 1 (1.2) |
| Intensive care unit (ICU) | 64 (5.0) | 7 (9.0) | 42 (8.9) | 1 (1.4) | 8 (36.4) | 12 (10.3) | 10 (11.8) |
| Clinic, laboratory, radiology department | 30 (2.3) | 4 (5.1) | 15 (3.2) | 3 (4.2) | 1 (4.5) | 7 (6.0) | 7 (8.2) |
| Operation room (OR) | 6 (0.5) | 0 (0.0) | 15 (3.2) | 1 (1.4) | 1 (4.5) | 1 (0.9) | 2 (2.4) |
| General ward | 279 (21.6) | 18 (23.1) | 177 (37.3) | 38 (53.5) | 7 (31.8) | 62 (53.0) | 19 (22.4) |

| **Supplement table S1:2** Basic characteristics presented in the 26 aetiologies of cardiac arrest | | | | | | | | |
| --- | --- | --- | --- | --- | --- | --- | --- | --- |
| **Cardiac or non-cardiac group** | **Non-cardiac group** | | | | | | | |
| **6 main aetiology categories** | **Infection** | | | **Pulmonary causes** | | | | **Other non-cardiac** |
| **26 aetiology categories** | **Other infection (%)** | **Septicaemia (%)** | **Asthma/**  **COPD (%)** | | **Respiratory insufficiency (%)** | **Aspiration (%)** | **Pulmonary embolism (%)** | **Non-categorized non-cardiac (%)** |
| n | 155 (3.6) | 234 (5.4) | 66 (1.5) | | 560 (13.0) | 97 (2.2) | 200 (4.6) | 84 (1.9) |
| Sex (Women) | 56 (36.1) | 83 (35.5) | 31 (47.0) | | 217 (38.8) | 42 (43.3) | 104 (52.0) | 27 (32.1) |
| Age (median [IQR]) | 74 [67, 83] | 73 [66, 81] | 74 [68, 79] | | 74 [65, 80] | 75 [68, 81] | 72 [64, 79] | 72 [62, 80] |
| **Comorbidity** |  |  |  | |  |  |  |  |
| Cancer | 58 (37.4) | 92 (39.3) | 22 (33.3) | | 237 (42.3) | 50 (51.5) | 77 (38.5) | 27 (32.1) |
| Diabetes | 58 (37.4) | 88 (37.6) | 17 (25.8) | | 171 (30.5) | 28 (28.9) | 49 (24.5) | 27 (32.1) |
| Renal failure | 39 (25.2) | 77 (32.9) | 14 (21.2) | | 128 (22.9) | 18 (18.6) | 23 (11.5) | 29 (34.5) |
| Cardiac failure | 63 (40.6) | 88 (37.6) | 27 (40.9) | | 215 (38.4) | 23 (23.7) | 40 (20.0) | 23 (27.4) |
| Myocardial infarction | 50 (32.3) | 65 (27.8) | 20 (30.3) | | 156 (27.9) | 15 (15.5) | 34 (17.0) | 27 (32.1) |
| Asthma/COPD | 30 (19.4) | 54 (23.1) | 62 (93.9) | | 154 (27.5) | 14 (14.4) | 26 (13.0) | 8 (9.5) |
| Addiction | 26 (16.8) | 40 (17.1) | 17 (25.8) | | 108 (19.3) | 14 (14.4) | 25 (12.5) | 21 (25.0) |
| Psychiatric comorbidity | 21 (13.5) | 30 (12.8) | 9 (13.6) | | 70 (12.5) | 11 (11.3) | 22 (11.0) | 4 (4.8) |
| Hypertension | 108 (69.7) | 159 (67.9) | 37 (56.1) | | 374 (66.8) | 60 (61.9) | 118 (59.0) | 55 (65.5) |
| **Location** |  |  |  | |  |  |  |  |
| Emergency department | 10 (6.5) | 24 (10.3) | 13 (19.7) | | 80 (14.3) | 9 (9.3) | 31 (15.5) | 9 (10.7) |
| Catheterization lab | 0 (0.0) | 0 (0.0) | 1 (1.5) | | 0 (0.0) | 1 (1.0) | 4 (2.0) | 1 (1.2) |
| Other | 2 (1.3) | 0 (0.0) | 1 (1.5) | | 8 (1.4) | 2 (2.1) | 5 (2.5) | 4 (4.8) |
| Cardiac care unit (CCU) | 3 (1.9) | 10 (4.3) | 4 (6.1) | | 42 (7.5) | 0 (0.0) | 17 (8.5) | 5 (6.0) |
| Intermediate care unit (IMCU) | 7 (4.5) | 18 (7.7) | 2 (3.0) | | 29 (5.2) | 3 (3.1) | 5 (2.5) | 1 (1.2) |
| Intensive care unit (ICU) | 13 (8.4) | 52 (22.2) | 1 (1.5) | | 53 (9.5) | 8 (8.2) | 25 (12.5) | 11 (13.1) |
| Clinic, laboratory, radiology department | 5 (3.2) | 11 (4.7) | 0 (0.0) | | 27 (4.8) | 3 (3.1) | 6 (3.0) | 6 (7.1) |
| Operation room (OR) | 1 (0.6) | 7 (3.0) | 0 (0.0) | | 10 (1.8) | 0 (0.0) | 6 (3.0) | 6 (7.1) |
| General ward | 114 (73.5) | 112 (47.9) | 44 (66.7) | | 311 (55.5) | 71 (73.2) | 101 (50.5) | 41 (48.8) |

| **Supplement table S1:3** Basic characteristics presented in the 26 aetiologies of cardiac arrest | | | | | | | |
| --- | --- | --- | --- | --- | --- | --- | --- |
| **Cardiac or non-cardiac group** | **Non-cardiac group** | | | | | | |
| **6 main aetiology categories** | **Other non-cardiac cause** | | | | | | |
| **26 aetiology categories** | **Anaphylaxis (%)** | **Acute abdomen (%)** | **Intoxication (%)** | **Suicide (%)** | **Cancer (%)** | **Neurological (%)** | **Electrolytic imbalance (%)** |
| n | 24 (0.6) | 87 (2.0) | 63 (1.5) | 35 (0.8) | 78 (1.8) | 93 (2.2) | 39 (0.9) |
| Sex (Women) | 15 (62.5) | 35 (40.2) | 35 (55.6) | 19 (54.3) | 40 (51.3) | 37 (39.8) | 19 (48.7) |
| Age (median [IQR]) | 66 [52, 76] | 76 [68, 80] | 55 [41, 70] | 34 [23, 59] | 72 [63, 76] | 76 [69, 82] | 72 [63, 75] |
| **Comorbidity** |  |  |  |  |  |  |  |
| Cancer | 13 (54.2) | 34 (39.1) | 13 (20.6) | 3 (8.6) | 70 (89.7) | 34 (36.6) | 14 (35.9) |
| Diabetes | 5 (20.8) | 31 (35.6) | 14 (22.2) | 3 (8.6) | 20 (25.6) | 27 (29.0) | 19 (48.7) |
| Renal failure | 3 (12.5) | 27 (31.0) | 7 (11.1) | 2 (5.7) | 15 (19.2) | 16 (17.2) | 15 (38.5) |
| Cardiac failure | 1 (4.2) | 32 (36.8) | 4 (6.3) | 0 (0.0) | 11 (14.1) | 19 (20.4) | 12 (30.8) |
| Myocardial infarction | 2 (8.3) | 22 (25.3) | 3 (4.8) | 0 (0.0) | 11 (14.1) | 24 (25.8) | 9 (23.1) |
| Asthma/COPD | 9 (37.5) | 18 (20.7) | 11 (17.5) | 5 (14.3) | 13 (16.7) | 16 (17.2) | 8 (20.5) |
| Addiction | 5 (20.8) | 14 (16.1) | 24 (38.1) | 20 (57.1) | 11 (14.1) | 13 (14.0) | 15 (38.5) |
| Psychiatric comorbidity | 7 (29.2) | 9 (10.3) | 17 (27.0) | 29 (82.9) | 3 (3.8) | 8 (8.6) | 9 (23.1) |
| Hypertension | 14 (58.3) | 59 (67.8) | 23 (36.5) | 4 (11.4) | 46 (59.0) | 60 (64.5) | 29 (74.4) |
| **Location** |  |  |  |  |  |  |  |
| Emergency department | 1 (4.2) | 10 (11.5) | 17 (27.0) | 3 (8.6) | 6 (7.7) | 15 (16.1) | 11 (28.2) |
| Catheterization lab | 1 (4.2) | 0 (0.0) | 1 (1.6) | 0 (0.0) | 0 (0.0) | 0 (0.0) | 1 (2.6) |
| Other | 0 (0.0) | 1 (1.1) | 4 (6.3) | 2 (5.7) | 2 (2.6) | 3 (3.2) | 0 (0.0) |
| Cardiac care unit (CCU) | 0 (0.0) | 2 (2.3) | 3 (4.8) | 0 (0.0) | 3 (3.8) | 3 (3.2) | 7 (17.9) |
| Intermediate care unit (IMCU) | 0 (0.0) | 2 (2.3) | 2 (3.2) | 0 (0.0) | 0 (0.0) | 5 (5.4) | 1 (2.6) |
| Intensive care unit (ICU) | 1 (4.2) | 9 (10.3) | 8 (12.7) | 2 (5.7) | 9 (11.5) | 3 (3.2) | 6 (15.4) |
| Clinic, laboratory, radiology department | 11 (45.8) | 3 (3.4) | 9 (14.3) | 1 (2.9) | 4 (5.1) | 10 (10.8) | 2 (5.1) |
| Operation room (OR) | 1 (4.2) | 8 (9.2) | 3 (4.8) | 0 (0.0) | 1 (1.3) | 0 (0.0) | 1 (2.6) |
| General ward | 9 (37.5) | 52 (59.8) | 16 (25.4) | 27 (77.1) | 53 (67.9) | 54 (58.1) | 10 (25.6) |

| **Supplement table S1:4** Basic characteristics presented in the 26 aetiologies of cardiac arrest | | | | | |
| --- | --- | --- | --- | --- | --- |
| **Cardiac or non-cardiac group** | **Non-cardiac group** | | **Excluded from further analysis** | | |
| **6 main aetiology categories** | **Haemorrhage** | |  |  |  |
| **26 aetiology categories** | **Aortic dissection (%)** | **Other bleeding (%)** | **Other (%)** | **Unknown (%)** | **Technical failure (%)** |
| n | 154 (3.6) | 213 (4.9) | 81 | 1501 | 4 |
| Sex (Women) | 67 (43.5) | 79 (37.1) | 36 (44.4) | 624 (41.6) | 0 (0.0) |
| Age (median [IQR]) | 76 [68, 80] | 74 [65, 80] | 74 [65, 81] | 75 [66, 82] | 70.5 [59, 74] |
| **Comorbidity** |  |  |  |  |  |
| Cancer | 42 (27.3) | 100 (46.9) | 34 (42.0) | 627 (41.8) | 2 (50.0) |
| Diabetes | 27 (17.5) | 58 (27.2) | 28 (34.6) | 466 (31.0) | 1 (25.0) |
| Renal failure | 30 (19.5) | 46 (21.6) | 24 (29.6) | 355 (23.7) | 0 (0.0) |
| Cardiac failure | 31 (20.1) | 47 (22.1) | 28 (34.6) | 526 (35) | 0 (0.0) |
| Myocardial infarction | 39 (25.3) | 51 (23.9) | 25 (30.9) | 517 (34.4) | 1 (25.0) |
| Asthma/COPD | 25 (16.2) | 26 (12.2) | 15 (18.5) | 312 (20.8) | 1 (25.0) |
| Addiction | 18 (11.7) | 40 (18.8) | 18 (22.2) | 228 (15.2) | 0 (0.0) |
| Psychiatric comorbidity | 11 (7.1) | 20 (9.4) | 10 (12.3) | 172 (11.5) | 0 (0.0) |
| Hypertension | 105 (68.2) | 130 (61.0) | 53 (65.4) | 970 (64.6) | 1 (25.0) |
| **Location** |  |  |  |  |  |
| Emergency department | 51 (33.1) | 38 (17.8) | 6 (7.4) | 166 (11.1) |  |
| Catheterization lab | 5 (3.2) | 2 (0.9) | 0 (0.0) | 95 (6.3) | 0 (0.0) |
| Other | 3 (1.9) | 1 (0.5) | 1 (1.2) | 48 (3.2) | 1 (25.0) |
| Cardiac care unit (CCU) | 12 (7.8) | 5 (2.3) | 5 (6.2) | 156 (10.4) | 0 (0.0) |
| Intermediate care unit (IMCU) | 3 (1.9) | 7 (3.3) | 4 (4.9) | 39 (2.6) | 0 (0.0) |
| Intensive care unit (ICU) | 6 (3.9) | 21 (9.9) | 11 (13.6) | 93 (6.2) | 1 (25.0) |
| Clinic, laboratory, radiology department | 22 (14.3) | 15 (7.0) | 5 (6.2) | 73 (4.9) | 1 (25.0) |
| Operation room (OR) | 0 (0.0) | 13 (6.1) | 3 (3.7) | 34 (2.3) | 1 (2.9) |
| General ward | 52 (33.8) | 111 (52.1) | 46 (56.8) | 797 (53.1) | 0 (0.0) |

| **Supplement table S2.1** Information of initial rhythm, circumstances, time, and treatment of IHCA presented in the 26 aetiologies of cardiac arrest | | | | | | | |
| --- | --- | --- | --- | --- | --- | --- | --- |
| **Cardiac or non-cardiac group** | **Cardiac group** | | | | | | |
| **6 aetiology categories** | **Myocardial ischemia** | **Other cardiac cause** | | | | | |
| **26 aetiology categories** | **Myocardial ischemia (%)** | **Cardiac tamponade (%)** | **Primary arrythmia (%)** | **Cardio-myopathy (%)** | **Arrythmia post cardiac surgery (%)** | **Cardiac failure (%)** | **Non-categorized cardiac (%)** |
| n | 1291 (29.6) | 78 (1.8) | 474 (11.0) | 71 (1.6) | 22 (0.5) | 117 (2.7) | 85 (2.0) |
| **Initial rhythm** |  |  |  |  |  |  |  |
| Asystole | 287 (24.6) | 27 (37.5) | 193 (46.4) | 20 (29.0) | 4 (21.1) | 37 (37.0) | 28 (35.4) |
| Pulseless electrical activity (PEA) | 300 (25.7) | 43 (59.7) | 33 (7.9) | 29 (42.0) | 3 (15.8) | 47 (47.0) | 17 (21.5) |
| Ventricular fibrillation (VF) | 443 (38.0) | 0 (0.0) | 93 (22.4) | 10 (14.5) | 8 (42.1) | 6 (6.0) | 26 (32.9) |
| Pulseless ventricular tachycardia (VT) | 137 (11.7) | 2 (2.8) | 97 (23.3) | 10 (14.5) | 4 (21.1) | 10 (10.0) | 8 (10.1) |
|  |  |  |  |  |  |  |  |
| Witnessed | 1170 (90.8) | 72 (92.3) | 424 (89.5) | 54 (76.1) | 20 (90.9) | 86 (74.1) | 79 (92.9) |
| ECG monitored | 1041 (81.6) | 61 (78.2) | 363 (77.1) | 39 (54.9) | 20 (90.9) | 54 (46.2) | 63 (77.8) |
| **Event times** |  |  |  |  |  |  |  |
| Recognition – call ≤1min | 835 (85.6) | 55 (84.6) | 338 (84.9) | 44 (73.3) | 11 (91.7) | 76 (78.4) | 49 (89.1) |
| Recognition – CPR ≤1min | 1068 (94.0) | 65 (87.8) | 405 (94.8) | 64 (91.4) | 16 (100.0) | 98 (91.6) | 64 (94.1) |
| Recognition – def ≤3min | 526 (80.6) | 2 (28.6) | 152 (80.4) | 18 (72.0) | 9 (81.8) | 17 (51.5) | 30 (93.8) |
| **Treatment at the scene of the arrest** |  |  |  |  |  |  |  |
| CPR started before RRT | 951 (90.2) | 65 (94.2) | 385 (93.0) | 62 (95.4) | 11 (91.7) | 96 (88.9) | 55 (93.2) |
| Chest compression | 894 (97.8) | 64 (100.0) | 367 (98.9) | 61 (100.0) | 11 (100.0) | 92 (100.0) | 50 (96.2) |
| Defibrillation performed | 694 (54.8) | 9 (12.0) | 204 (44.3) | 28 (39.4) | 14 (66.7) | 38 (33.0) | 38 (44.7) |
| Intubation | 547 (43.3) | 50 (67.6) | 93 (20.2) | 35 (49.3) | 7 (35.0) | 69 (60.0) | 30 (35.7) |
| Epinephrine | 734 (57.9) | 66 (88.0) | 154 (33.5) | 51 (71.8) | 7 (31.8) | 97 (82.9) | 43 (51.2) |
| **Post cardiac arrest treatment** |  |  |  |  |  |  |  |
| PCI | 492 (59.9) | 7 (20.6) | 31 (7.6) | 2 (5.4) | 2 (10.0) | 1 (2.7) | 11 (16.9) |
| ICD | 44 (5.4) | 0 (0.0) | 68 (16.7) | 5 (13.5) | 5 (25.0) | 0 (0.0) | 8 (12.3) |
| Pacemaker during hospital stay | 78 (9.5) | 3 (8.8) | 202 (49.6) | 6 (16.2) | 8 (40.0) | 2 (5.4) | 15 (23.1) |
| Coronary angiography | 493 (60.0) | 9 (26.5) | 87 (21.4) | 10 (27.0) | 5 (25.0) | 3 (8.1) | 23 (35.4) |
| CABG during hospital stay | 24 (2.9) | 1 (2.9) | 2 (0.5) | 0 (0.0) | 0 (0.0) | 1 (2.7) | 0 (0.0) |

| **Supplement table S2.2** Information of initial rhythm, circumstances, time, and treatment presented in the 26 aetiologies of cardiac arrest | | | | | | | | |
| --- | --- | --- | --- | --- | --- | --- | --- | --- |
| **Cardiac or non-cardiac group** | **Non-cardiac group** | | | | | | | |
| **6 aetiology categories** | **Infection** | | | **Pulmonary causes** | | | | **Other non-cardiac cause** |
| **26 aetiology categories** | **Other infection (%)** | **Septicaemia (%)** | **Asthma/**  **COPD (%)** | | **Respiratory insufficiency (%)** | **Aspiration (%)** | **Pulmonary embolism (%)** | **Non-categoriezed non-cardiac (%)** |
| n | 155 (3.6) | 234 (5.4) | 66 (1.5) | | 560 (13.0) | 97 (2.2) | 200 (4.6) | 84 (1.9) |
| **Initial rhythm** |  |  |  | |  |  |  |  |
| Asystole | 58 (47.5) | 101 (52.9) | 27 (50.0) | | 206 (49.4) | 34 (46.6) | 56 (33.7) | 39 (55.7) |
| Pulseless electrical activity (PEA) | 53 (43.4) | 69 (36.1) | 23 (42.6) | | 186 (44.6) | 35 (47.9) | 99 (59.6) | 23 (32.9) |
| Ventricular fibrillation (VF) | 10 (8.2) | 14 (7.3) | 1 (1.9) | | 14 (3.4) | 3 (4.1) | 5 (3.0) | 6 (8.6) |
| Pulseless ventricular tachycardia (VT) | 1 (0.8) | 7 (3.7) | 3 (5.6) | | 11 (2.6) | 1 (1.4) | 6 (3.6) | 2 (2.9) |
|  |  |  |  | |  |  |  |  |
| Witnessed | 98 (64.5) | 192 (82.8) | 48 (73.8) | | 436 (78.4) | 79 (81.4) | 171 (85.5) | 66 (78.6) |
| ECG monitored | 46 (30.1) | 132 (57.1) | 22 (33.3) | | 231 (42.4) | 27 (28.7) | 107 (54.9) | 42 (51.2) |
| **Event times** |  |  |  | |  |  |  |  |
| Recognition – call ≤1min | 101 (77.1) | 148 (83.1) | 45 (76.3) | | 362 (78.4) | 56 (70.9) | 119 (76.8) | 59 (85.5) |
| Recognition – CPR ≤1min | 136 (92.5) | 199 (89.6) | 53 (88.3) | | 475 (90.8) | 76 (83.5) | 172 (92.0) | 70 (88.6) |
| Recognition – def ≤3min | 9 (45.0) | 16 (34.0) | 2 (25.0) | | 28 (45.9) | 4 (28.6) | 9 (27.3) | 6 (37.5) |
| **Treatment at the scene of the arrest** |  |  |  | |  |  |  |  |
| CPR started before RRT | 132 (95.7) | 178 (93.7) | 61 (95.3) | | 449 (91.8) | 83 (94.3) | 160 (92.0) | 68 (91.9) |
| Chest compression | 131 (100.0) | 169 (99.4) | 60 (100.0) | | 431 (99.1) | 80 (100.0) | 157 (100.0) | 66 (100.0) |
| Defibrillation performed | 25 (16.7) | 51 (22.6) | 10 (15.4) | | 58 (10.7) | 14 (14.9) | 35 (17.7) | 20 (25.3) |
| Intubation | 79 (52.3) | 151 (65.7) | 28 (45.2) | | 324 (59.9) | 66 (69.5) | 138 (69.7) | 49 (60.5) |
| Epinephrine | 116 (77.3) | 195 (83.3) | 46 (71.9) | | 410 (75.4) | 78 (82.1) | 173 (86.5) | 60 (73.2) |
| **Post cardiac arrest treatment** |  |  |  | |  |  |  |  |
| PCI | 0 (0.0) | 1 (0.8) | 1 (2.4) | | 2 (0.6) | 0 (0.0) | 1 (1.0) | 2 (4.3) |
| ICD | 0 (0.0) | 0 (0.0) | 0 (0.0) | | 2 (0.6) | 0 (0.0) | 0 (0.0) | 1 (2.1) |
| Pacemaker during hospital stay | 1 (1.6) | 5 (3.8) | 1 (2.4) | | 6 (1.8) | 0 (0.0) | 0 (0.0) | 1 (2.1) |
| Coronary angiography | 1 (1.6) | 1 (0.8) | 0 (0.0) | | 11 (3.2) | 0 (0.0) | 7 (7.2) | 4 (8.5) |
| CABG during hospital stay | 0 (0.0) | 0 (0.0) | 0 (0.0) | | 2 (0.6) | 0 (0.0) | 0 (0.0) | 0 (0.0) |

| **Supplement table S2.3** Information of initial rhythm, circumstances, time, and treatment presented in the 26 aetiologies of cardiac arrest | | | | | | | |
| --- | --- | --- | --- | --- | --- | --- | --- |
| **Cardiac or non-cardiac group** | **Non-cardiac group** | | | | | | |
| **6 aetiology categories** | **Other non-cardiac cause** | | | | | | |
| **26 aetiology categories** | **Anaphylaxis (%)** | **Acute abdomen (%)** | **Intoxication (%)** | **Suicide (%)** | **Cancer (%)** | **Neurological (%)** | **Electrolytic imbalance (%)** |
| n | 24 (0.6) | 87 (2.0) | 63 (1.5) | 35 (0.8) | 78 (1.8) | 93 (2.2) | 39 (0.9) |
| **Initial rhythm** |  |  |  |  |  |  |  |
| Asystole | 3 (20.0) | 35 (47.9) | 29 (58.0) | 16 (59.3) | 34 (54.0) | 30 (44.1) | 6 (20.0) |
| Pulseless electrical activity (PEA) | 11 (73.3) | 30 (41.1) | 11 (22.0) | 9 (33.3) | 23 (36.5) | 29 (42.6) | 8 (26.7) |
| Ventricular fibrillation (VF) | 1 (6.7) | 4 (5.5) | 7 (14.0) | 2 (7.4) | 3 (4.8) | 6 (8.8) | 7 (23.3) |
| Pulseless ventricular tachycardia (VT) | 0 (0.0) | 4 (5.5) | 3 (6.0) | 0 (0.0) | 3 (4.8) | 3 (4.4) | 9 (30.0) |
|  |  |  |  |  |  |  |  |
| Witnessed | 23 (95.8) | 70 (80.5) | 54 (85.7) | 8 (23.5) | 53 (68.8) | 72 (77.4) | 37 (94.9) |
| ECG monitored | 8 (33.3) | 36 (41.4) | 39 (61.9) | 5 (14.7) | 20 (26.7) | 41 (44.6) | 23 (60.5) |
| **Event times** |  |  |  |  |  |  |  |
| Recognition – call ≤1min | 17 (85.0) | 60 (81.1) | 39 (79.6) | 16 (53.3) | 46 (67.6) | 59 (81.9) | 19 (70.4) |
| Recognition – CPR ≤1min | 18 (94.7) | 74 (88.1) | 55 (91.7) | 26 (76.5) | 64 (83.1) | 70 (86.4) | 31 (93.9) |
| Recognition – def ≤3min | 0 (0.0) | 4 (26.7) | 8 (61.5) | 3 (50.0) | 5 (38.5) | 5 (33.3) | 11 (84.6) |
| **Treatment at the scene of the arrest** |  |  |  |  |  |  |  |
| CPR started before RRT | 16 (66.7) | 72 (93.5) | 49 (94.2) | 29 (93.5) | 63 (91.3) | 75 (89.3) | 27 (93.1) |
| Chest compression | 16 (100.0) | 69 (100.0) | 49 (100.0) | 29 (100.0) | 60 (100.0) | 73 (97.3) | 25 (100.0) |
| Defibrillation performed | 2 (9.1) | 15 (17.2) | 14 (22.6) | 5 (14.3) | 15 (19.7) | 17 (18.7) | 18 (47.4) |
| Intubation | 14 (63.6) | 55 (64.0) | 28 (45.9) | 24 (68.6) | 38 (49.4) | 45 (50.6) | 16 (42.1) |
| Epinephrine | 16 (72.7) | 74 (85.1) | 28 (46.7) | 24 (70.6) | 54 (72.0) | 55 (61.1) | 16 (44.4) |
| **Post cardiac arrest treatment** |  |  |  |  |  |  |  |
| PCI | 1 (4.3) | 1 (2.2) | 0 (0.0) | 0 (0.0) | 0 (0.0) | 1 (1.7) | 1 (2.9) |
| ICD | 0 (0.0) | 0 (0.0) | 0 (0.0) | 0 (0.0) | 0 (0.0) | 2 (3.3) | 0 (0.0) |
| Pacemaker during hospital stay | 1 (4.3) | 1 (2.2) | 6 (11.3) | 0 (0.0) | 1 (3.8) | 3 (5.0) | 2 (5.9) |
| Coronary angiography | 2 (8.7) | 3 (6.5) | 6 (11.3) | 0 (0.0) | 1 (3.8) | 3 (5.0) | 6 (17.6) |
| CABG during hospital stay | 0 (0.0) | 0 (0.0) | 0 (0.0) | 0 (0.0) | 0 (0.0) | 0 (0.0) | 0 (0.0) |

| **Supplement table S2.4** Information of initial rhythm, circumstances, time, and treatment presented in the 26 aetiologies of cardiac arrest | | | | | |
| --- | --- | --- | --- | --- | --- |
| **Cardiac or non-cardiac group** | **Non-cardiac group** | | **Excluded from further analysis** | | |
| **6 aetiology categories** | **Haemorrhage** | |  |  |  |
| **26 aetiology categories** | **Aortic dissection (%)** | **Other bleeding (%)** | **Other (%)** | **Unknown (%)** | **Technical failure (%)** |
| n | 154 (3.6) | 213 (4.9) | 81 | \| 1501 \| \| --- \| | 4 |
| **Initial rhythm** |  |  |  |  |  |
| Asystole | 40 (29.9) | 80 (51.3) | 27 (40.9) | 522 (47.4) | 1 (25.0) |
| Pulseless electrical activity (PEA) | 88 (65.7) | 64 (41.0) | 23 (34.8) | 365 (33.2) | 2 (50.0) |
| Ventricular fibrillation (VF) | 6 (4.5) | 8 (5.1) | 8 (12.1) | 137 (12.4) | 1 (25.0) |
| Pulseless ventricular tachycardia (VT) | 0 (0.0) | 4 (2.6) | 8 (12.1) | 77 (7.0) | 0 (0.0) |
|  |  |  |  |  |  |
| Witnessed | 124 (81.0) | 170 (80.6) | 54 (68.4) | 1079 (72.6) | 4 (100.0) |
| ECG monitored | 86 (57.3) | 88 (42.1) | 36 (45.6) | 655 (44.3) | 3 (100.0) |
| **Event times** |  |  |  |  |  |
| Recognition – call ≤1min | 121 (89.0) | 141 (80.6) | 48 (72.7) | 954 (75.9) | 2 (100.0) |
| Recognition – CPR ≤1min | 138 (92.0) | 178 (90.8) | 68 (93.2) | 1201 (87.9) | 4 (100.0) |
| Recognition – def ≤3min | 7 (30.4) | 10 (33.3) | 12 (57.1) | 201 (61.1) | 1 (50.0) |
| **Treatment at the scene of the arrest** |  |  |  |  |  |
| CPR started before RRT | 129 (90.2) | 170 (90.9) | 65 (92.9) | 1204 (91.7) | 2 (100.0) |
| Chest compression | 127 (100.0) | 161 (98.2) | 62 (98.4) | 1163 (99.5) | 2 (100.0) |
| Defibrillation performed | \| 28 (18.4) \| \| --- \| | \| 31 (14.8) \| \| --- \| | \| 19 (25.3) \| \| --- \| | 363 (25.4) | 3 (75.0) |
| Intubation | 107 (70.9) | 129 (62.9) | 38 (49.4) | 710 (50.1) | 2 (50.0) |
| Epinephrine | 131 (86.8) | 153 (72.9) | 55 (71.4) | 966 (67.4) | 3 (75.0) |
| **Post cardiac arrest treatment** |  |  |  |  |  |
| PCI | 1 (2.4) | 1 (1.0) | 0 (0.0) | 4 (1.1) | 0 (0.0) |
| ICD | 0 (0.0) | 0 (0.0) | 1 (2.1) | 10 (2.7) | 0 (0.0) |
| Pacemaker during hospital stay | 1 (2.4) | 0 (0.0) | 3 (6.2) | 16 (4.3) | 1 (25.0) |
| Coronary angiography | 1 (2.4) | 2 (1.9) | 2 (4.2) | 22 (5.9) | 0 (0.0) |
| CABG during hospital stay | 0 (0.0) | 0 (0.0) | 0 (0.0) | 1 (0.3) | 0 (0.0) |

| **Supplement table S3.1** Outcome of IHCA presented in the 26 aetiologies of cardiac arrest | | | | | | | |
| --- | --- | --- | --- | --- | --- | --- | --- |
| **Cardiac or non-cardiac group** | **Cardiac group** | | | | | | |
| **6 aetiology categories** | **Myocardial ischemia** | **Other cardiac cause** | | | | | |
| **26 aetiology categories** | **Myocardial ischemia (%)** | **Cardiac tamponade (%)** | **Primary arrythmia (%)** | **Cardio-**  **Myopathy (%)** | **Arrythmia post cardiac surgery (%)** | **Cardiac failure (%)** | **Non-categorized cardiac (%)** |
| n | 1291 (29.9) | 78 (1.8) | 474 (11.0) | 71 (1.6) | 22 (0.5) | 117 (2.7) | 85 (2.0) |
| ROSC | 784 (61.5) | 27 (35.5) | 397 (84.8) | 37 (52.1) | 19 (86.4) | 33 (28.4) | 64 (76.2) |
| 30-day survival | 565 (43.8) | 19 (24.4) | 331 (69.8) | 24 (33.8) | 15 (68.2) | 10 (8.5) | 54 (63.5) |
| **CPC** |  |  |  |  |  |  |  |
| CPC 1-2 | 515 (91.2) | 11 (61.1) | 290 (89.2) | 20 (87.0) | 13 (81.2) | 5 (55.6) | 46 (92.0) |
| CPC 3-5 | 18 (3.2) | 4 (22.2) | 15 (4.6) | 0 (0.0) | 1 (6.2) | 2 (22.2) | 2 (4.0) |
| Unknown | 32 (5.7) | 3 (16.7) | 20 (6.2) | 3 (13.0) | 2 (12.5) | 2 (22.2) | 2 (4.0) |

| **Supplement table S3.2** Outcome of IHCA presented in the 26 aetiologies of cardiac arrest | | | | | | | |
| --- | --- | --- | --- | --- | --- | --- | --- |
| **Cardiac or non-cardiac group** | **Non-cardiac group** | | | | | | |
| **6 aetiology categories** | **Infection** | | **Pulmonary causes** | | | | **Other non-cardiac cause** |
| **26 aetiology categories** | **Other infection (%)** | **Septicaemia (%)** | **Asthma/**  **COPD (%)** | **Respiratory insufficiency (%)** | **Aspiration (%)** | **Pulmonary embolism (%)** | **Non-categoriezed non-cardiac (%)** |
| n | 155 (3.6) | 234 (5.4) | 66 (1.5) | 560 (13.0) | 97 (2.2) | 200 (4.6) | 84 (1.9) |
| ROSC | 58 (37.4) | 126 (54.3) | 38 (60.3) | 323 (58.7) | 54 (56.8) | 88 (44.4) | 43 (53.1) |
| 30-day survival | 26 (16.8) | 37 (15.8) | 10 (15.2) | 141 (25.2) | 13 (13.4) | 39 (19.5) | 28 (33.3) |
| **CPC** |  |  |  |  |  |  |  |
| CPC 1-2 | 12 (52.2) | 20 (55.6) | 10 (76.9) | 95 (72.0) | 6 (66.7) | 27 (73.0) | 16 (66.7) |
| CPC 3-5 | 6 (26.1) | 12 (33.3) | 2 (15.4) | 30 (22.7) | 2 (22.2) | 9 (24.3) | 7 (29.2) |
| Unknown | 5 (21.7) | 4 (11.1) | 1 (7.7) | 7 (5.3) | 1 (11.1) | 1 (2.7) | 1 (4.2) |

| **Supplement table S3.3** Outcome of IHCA presented in the 26 aetiologies of cardiac arrest | | | | | | | |
| --- | --- | --- | --- | --- | --- | --- | --- |
| **Cardiac or non-cardiac group** | **Non-cardiac group** | | | | | | |
| **6 aetiology categories** | **Other non-cardiac cause** | | | | | | |
| **26 aetiology categories** | **Anaphylaxis (%)** | **Acute abdomen (%)** | **Intoxication (%)** | **Suicide (%)** | **Cancer (%)** | **Neurological (%)** | **Electrolytic imbalance (%)** |
| n | 24 (0.6) | 87 (2.0) | 63 (1.5) | 35 (0.8) | 78 (1.8) | 93 (2.2) | 39 (0.9) |
| ROSC | 21 (95.5) | 44 (51.2) | 53 (85.5) | 21 (60.0) | 21 (27.3) | 55 (59.8) | 33 (86.8) |
| 30-day survival | 18 (75.0) | 11 (12.6) | 47 (74.6) | 7 (20.0) | 2 (2.6) | 14 (15.1) | 25 (64.1) |
| **CPC** |  |  |  |  |  |  |  |
| CPC 1-2 | 15 (88.2) | 7 (70.0) | 33 (80.5) | 6 (85.7) | 2 (100.0) | 6 (42.9) | 22 (88.0) |
| CPC 3-5 | 0 (0.0) | 2 (20.0) | 5 (12.2) | 1 (14.3) | 0 (0.0) | 6 (42.9) | 2 8.0) |
| Unknown | 2 (11.8) | 1 (10.0) | 3 (7.3) | 0 (0.0) | 0 (0.0) | 2 (14.3) | 1 (4.0) |

| **Supplement table S3.4** Outcome of IHCA presented in the 26 aetiologies of cardiac arrest | | | | | |
| --- | --- | --- | --- | --- | --- |
| **Cardiac or non-cardiac group** | **Non-cardiac group** | | **Excluded from further analysis** | | |
| **6 aetiology categories** | **Haemorrhage** | |  |  |  |
| **26 aetiology categories** | **Aortic dissection (%)** | **Other bleeding (%)** | **Other (%)** | **Unknown (%)** | **Technical failure (%)** |
| n | 154 (3.6) | 213 (4.9) | 81 | 852 | 4 |
| ROSC | 31 (20.4) | 99 (46.9) | 44 (57.1) | 340 (40.7) | 4 (100.0) |
| 30-day survival | 6 (3.9) | 48 (22.5) | 23 (28.4) | 151 (17.7) | 3 (75.0) |
| **CPC** |  |  |  |  |  |
| CPC 1-2 | 6 (100.0) | 38 (86.4) | 13 (65.0) | 99 (69.2) | 2 (66.7) |
| CPC 3-5 | 0 (0.0) | 4 (9.1) | 4 (20.0) | 20 (14.0) | 0 (0.0) |
| Unknown | 0 (0.0) | 2 (4.5) | 3 (15) | 24 (16.8) | 1 (33.3) |

| **Supplement table S4.** Outcomes in monitored and unmonitored patients | | | |
| --- | --- | --- | --- |
|  | **Unmonitored** (%) | **Monitored** (%) | **P-value** |
| n | 2527 | 3288 |  |
| ROSC (%) | 1108 (44.6) | 2066 (63.9) | <0.001 |
| 30-day survival (%) | 526 (20.8) | 1380 (42.0) | <0.001 |
| **CPC (%)** |  |  | 0.006 |
| CPC 1-2 | 328 (78.5) | 993 (84.5) |  |
| CPC 3-5 | 55 (13.2) | 95 ( 8.1) |  |
| Unknown CPC | 35 ( 8.4) | 87 ( 7.4) |  |

| **Supplement figure S1.** Explanation of aetiologies reported to the Swedish registry of cardiopulmonary resuscitation | |
| --- | --- |
| **Aetiology** | **Explanation** |
| Anaphylactic shock | Shock caused by allergy or hypersensitivity |
| Asthma/COPD | Obstructive lung disease |
| Aspiration | Gastric content that has entered the airways |
| Other respiratory insufficiency | Respiratory failure due to other causes than aspiration, asthma, or COPD |
| Myocardial ischemia | Insufficient blood supply to parts of the heart. E.g., acute myocardial infarction or unstable angina pectoris |
| Primary arrythmia | Ventricular fibrillation or other arrythmia without known underlying heart disease |
| Arrythmia post cardiac surgery |  |
| Cardiomyopathy | Hypertrophic or dilated cardiomyopathy |
| Stroke | Part of the brain´s nerve cells have been damage or died due to insufficient blood flow in the brain’s vessels, caused by bleeding or infarction. |
| Pulmonary thromboembolism |  |
| Aortic dissection/rupture | Rupture of the inner aortic wall that either leads to bleeding within or rupture of the aortic wall. |
| Tamponade | The pericardium fills with blood or fluid |
| Other bleeding | Bleeding apart from intracerebral bleeding, tamponade, and aortic dissection. E.g., gastric haemorrhage or subdural hematoma. |
| Acute abdomen | All diagnoses of acute abdomen such as appendicitis, biliary disorders, and ileus. With the exception of gastric haemorrhage |
| Sepsis | Diagnosed sepsis |
| Other infection | Infection not fulfilling the diagnosis of sepsis. E.g., pneumonia, or urinary tract infection |
| Cancer | All types of cancer regardless of metastasized or not. |
| Intoxication | Excessive intake, accidental or intentional, of a drug or harmful substance |
| Suicide | Suicide or attempted suicide, except for intoxication |
| Technical failure | Technical failure of device. E.g., ventilator failure |
| Unknown | The cause of the cardiac arrest is unknown |
| Other | Option to fill in the cause of the cardiac arrest in free text. |
|  | |

**Supplement figure S2.** Frequency distribution of aetiologies of cardiac arrest caused by non-cardiac causes.

**Supplement figure S3.** Frequency distribution of aetiologies of cardiac arrest caused by cardiac causes.

**Suppelment figure S4.** Complete case analysis. Forrest plot of 30-day survival. Model 1, unadjusted 30-day survival. Model 2, 30-day survival adjusted for confounders and intermediates.


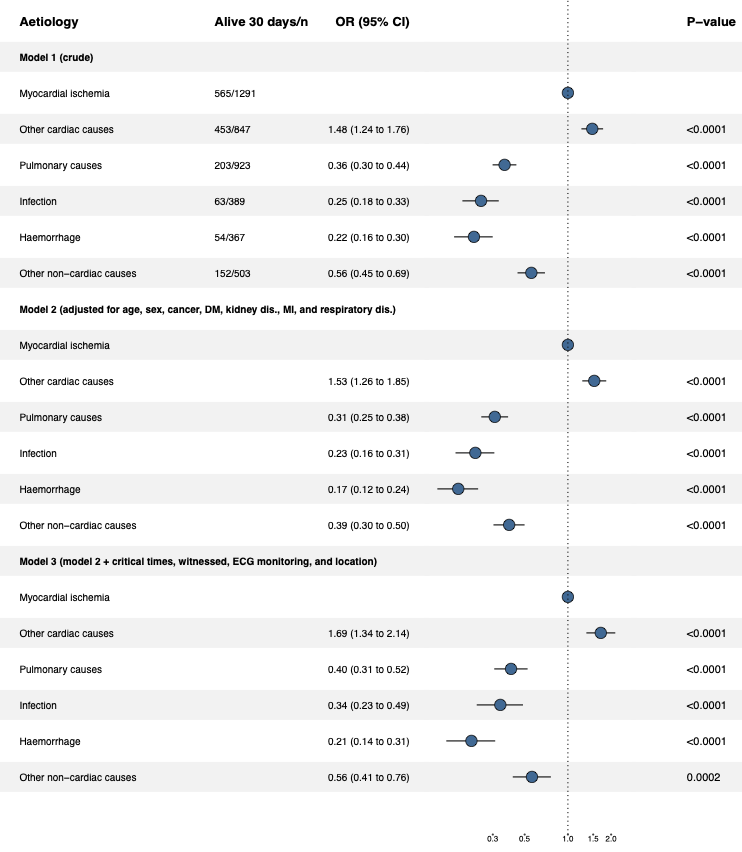


**Suppelment figure S5.** Complete case analysis. Forrest plot of ROSC. Model 1, unadjusted ROSC. Model 2, 30-day survival adjusted for confounders and intermediates.


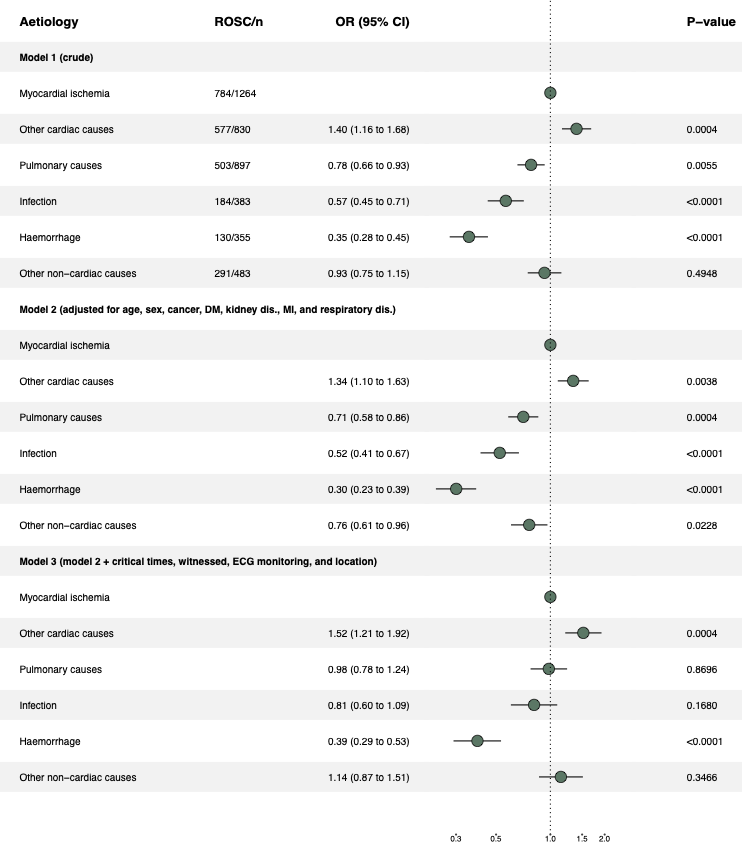


**Suppelment figure S6.** Complete case analysis. Forrest plot of favourable neurological outcome among survivors (CPC score 1-2). Model 1, unadjusted CPC-score 1-2. .Model 2, 30-day survival adjusted for confounders and intermediates.


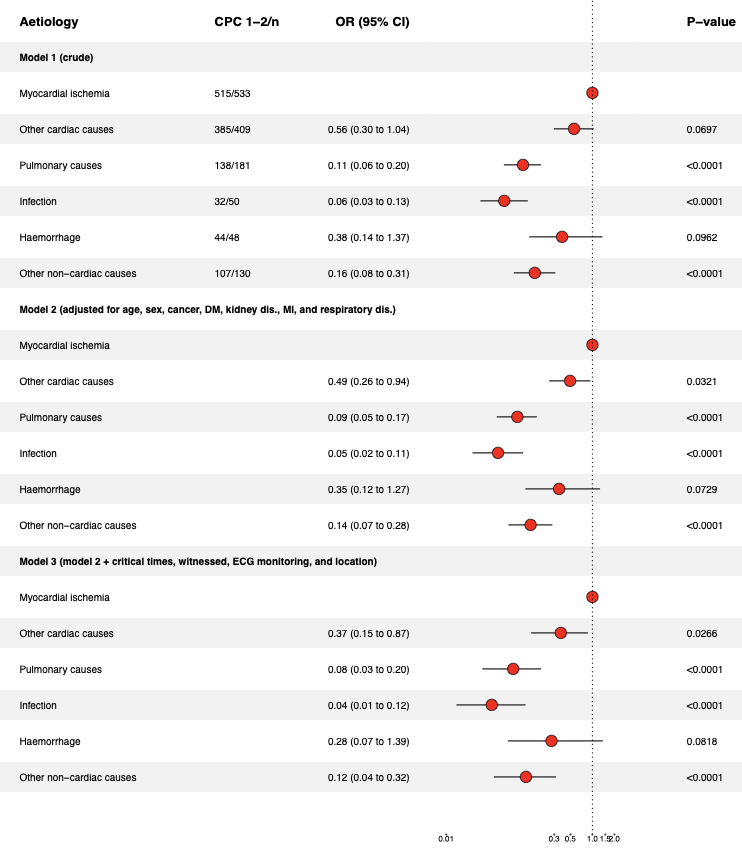

Supplement: oead066_Supplementary_Data [file oead066_supplementary_data.docx]
